# Antibody targeting TDP-43 mitigates pathogenic pathways induced by the cerebrospinal fluid of ALS

**DOI:** 10.1016/j.neurot.2025.e00737

**Published:** 2025-09-11

**Authors:** Amélie Poulin-Brière, Silvia Pozzi, Jean-Pierre Julien

**Affiliations:** a CERVO Brain Research Center 2601 Chemin de la Canardière, Québec, QC G1J 2G3, Canada; b Department of Psychiatry and Neuroscience, Laval University, Québec, QC G1V 0A6, Canada

**Keywords:** Sporadic ALS, Therapy, Antibody, TDP-43, CSF

## Abstract

Amyotrophic lateral sclerosis (ALS) is an incurable neurodegenerative disease characterized by the cytoplasmic mislocalization and accumulation of TAR DNA binding protein 43 (TDP-43). We reported previously the protective effects in a transgenic mouse model expressing ALS-linked mutant TDP-43^A315T^ of a monoclonal antibody, called E6, binding specifically to the RNA Recognition Motif 1 (RRM1) domain of TDP-43. Here, we tested the effects of E6 antibody in an animal model of sporadic ALS based on the intracerebroventricular (i.c.v.) infusion during 14 days of cerebrospinal fluid (CSF) from sporadic ALS patients into transgenic mice expressing human TDP-43^WT^. Either intrathecal (i.t.) or i.c.v. injection of E6 antibody conferred protective effects in this model of disease. Thus, the CSF-inoculated E6 antibody reduced motor and cognitive impairments, mitigated TDP-43 proteinopathy and prevented neurofilament (Nf) disorganization in cortical and spinal neurons. Administration of E6 antibody reduced the loss of motor neurons in the spinal cord and the denervation of neuromuscular junctions. Moreover, E6 antibody promoted a switch toward features associated with a protective phenotype of microglial activation characterized by enhanced phagocytic function and reduced secretion of pro-inflammatory cytokines. The results suggest that an immunotherapy targeting the RRM1 domain of TDP-43 may confer protection against pathogenic pathways triggered by the CSF of ALS patients.

## Graphical abstract

**Figure:**
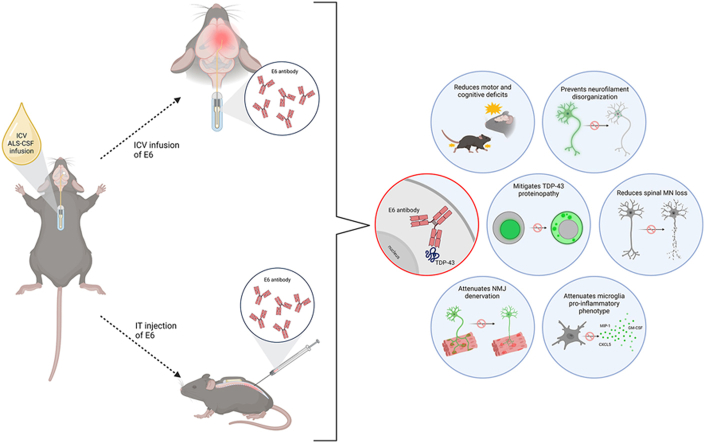

## Introduction

TDP-43 proteinopathy is a hallmark of ALS as well as of other neurodegenerative diseases such as frontotemporal lobar degeneration and Alzheimer disease [1,2]. TDP-43 proteinopathy is characterized by the mislocalization of the protein from the nucleus to the cytoplasm of cells, mainly neurons, the accumulation of the protein in the cytoplasm and the formations of aggregates, as well as by the cleavage of the protein into pathological fragments [3]. These events lead to a loss of normal nuclear function, due to the absence of functional TDP-43 in the nucleus, and to a gain of toxic function due to the presence of these aggregates and pathological fragments [4,5]. Moreover, several studies have highlighted the prion-like properties of TDP-43, allowing the TDP-43 proteinopathy to spread to healthy neighboring cells [[6], [7], [8]].

In the last fifteen years, many studies have tested the efficacy of antibody-based therapies targeting various proteins involved in ALS, with promising results, suggesting the suitability of immunotherapies for the treatment of ALS [9]. Recently, our group demonstrated the therapeutic efficacy of a monoclonal full-length antibody targeting TDP-43 called E6 and E6-derived-single-chain antibodies in transgenic mice expressing fALS-linked mutant TDP-43^A315T^ [9]. Indeed, these antibodies promoted TDP-43 degradation*,* reducing TDP-43 cytoplasmic mislocalization and aggregation, as well as improved motor and cognitive performance of TDP-43^A315T^ mice. Although promising, these results are applicable only to a small portion of ALS cases as fALS represent only 10 ​% of ALS cases in which only 4–5 ​% involve TDP-43 mutations. Although sALS represents 90 ​% of ALS cases [10], very few sALS models are available to study the disease. The exact causes and mechanisms of sALS still remain unclear. However, several studies have demonstrated the toxicity of CSF from ALS patients toward cultured cells and animals [[11], [12], [13], [14], [15], [16], [17], [18], [19], [20], [21]], suggesting that the CSF is involved in the pathophysiology of the disease as well as in its propagation. In 2015, it was hypothesized that toxic factors present in the CSF, such as SOD1, Fused in Sarcoma (FUS) and TDP-43 proteins, may be involved in the spreading of ALS [22].

Few years ago, our group reported that the i.c.v. infusion of CSF from sALS patients in mice expressing human TDP-43 wild type (hTDP-43^WT^) induced within 14 days ALS-like phenotypes, including motor and cognitive deficits, TDP-43 proteinopathy, Nf disorganization and neuroinflammation [15]. Here, we report that i.t. or i.c.v. delivery of the E6 antibody targeting TDP-43 alleviated multiple pathogenic pathways triggered by infusion of sALS-CSF in transgenic hTDP-43^WT^ mice. Our results suggest for the first time that TDP-43 is a key player in the pathogenesis triggered by the toxicity of CSF from ALS patients.

## Material and methods

### Study design

We examined both male and female animals, and no sex-dependent difference was observed for behavioral tests. Male tissues were used for biochemical assays and female tissues were used for immunofluorescence to reduce variability within groups.

### CSF samples and study groups

CSF samples were collected from ALS patients diagnosed with sporadic ALS (ALS-CSF, n ​= ​25) based on the revised El-Escorial criteria [23], after obtaining informed consent in accordance with the institutional human ethics committee guidelines (Approval 2022–2363). Human CSF samples from both sexes were used to prepare CSF pools. A first pool of ALS-CSF samples (n ​= ​9) was prepared and used to investigate the effects of the antibody after i.t. delivery. A second pool of ALS-CSF samples (n ​= ​25) was prepared by adding 16 new ALS-CSF samples to the previous 9 used in the first pool. This new pool was used to investigate the effects of the antibody after i.c.v. delivery. The 25 samples included CSF from 18 men and 7 women with a mean age of 59,2 ​± ​2,5 years (Table 1). CSF samples from age-matched subjects with normal pressure hydrocephalus (n ​= ​3) pathology or elevated intracranial pressure (n ​= ​7) were also collected and studied as non-ALS disease control (NALS-CSF, n ​= ​10, mean age 50,8 ​± ​7,5 years) for the study of i.c.v. treatment. After collection, CSF samples were centrifuged 10 ​min at 2000g at 4 ​°C before being snap frozen and kept in liquid nitrogen until use.

**Table 1:** Characteristics of CSF samples.

	ALS	NALS	P-value
N (Male/Female)	25 (18/6)	10 (5/4)	–
Age (years)	59.16 (29–85)	50.78 (23–81)	0.177
Protein concentration	2.22 (0.92–4.44)	2.62 (1.26–4.16)	0.209

Data are expressed as mean (min-max). ALS, amyotrophic lateral sclerosis; NALS, Non-ALS control (Normal Pressure Hydrocephalus and Elevated Intracranial Pressure).

For the present study, ALS- and NALS-CSF samples were separately pooled. CSF samples were thawed on ice. Pools were prepared by mixing individual samples to equal amount of proteins and were aliquoted and kept at −80 ​°C until use. ALS-CSF was used to induce the pathology by i.c.v. infusion. Then, in these mice, the therapeutic effects of an anti-TDP-43 antibody on ALS–CSF–induced pathology was evaluated after i.t. administration or i.c.v. administration of the antibody.

TDP-43 protein levels were measured in individual CSF samples using AlphaLISA Human TDP-43 Detection kit from PerkinElmer (catalog #AL387HV). ApoB protein levels were measured in individuals CSF samples using Human ApoB ELISA Kit from Invitrogen (catalog # EH34RB) according to manufacturer instructions.

### Mouse genotyping, surgical procedure and CSF administration

The hTDP-43^WT^ (heterozygous) mice [40] were identified by PCR on DNA from ear biopsies using forward and reverse primers 5′- GGATGAGCTGCAGTTCT-3′ and 5′-TGCCCATCAT ACCCCAACTG-3’ and constantly maintained, after more than 20 backcrosses, on a C57BL/6 strain (Charles River Laboratories) as a colony at the CERVO Brain Research Center animal facility under standard conditions. The antibody therapeutic efficacy was assessed in 8-month-old hTDP-43^WT^ mice (*n* ​= ​40 in total; *n* ​= ​5 males and *n* ​= ​5 females per group). Treatment randomization was applied, and mouse groups were composed at sex parity.

Osmotic pump (model 1002; Alzet) were filled with 100 ​μl of CSF pool and then incubated overnight in sterile PBS at 37 ​°C before implantation. For all surgical procedures, mice were anesthetized with 2.5 ​% isoflurane. For i.c.v. infusion of CSF, mice were pretreated with lidocaine-bupivacaine and slow-release buprenorphine to prevent postoperative pain. Mice were placed in a stereotaxic apparatus (David Kopf Instruments), and the right ventricle was then reached (1.50 ​mm lateral, −1.00 ​mm antero-posterior from Bregma) with a 33-gauge stainless steel cannula (Alzet) that was connected to the pump with an intramedic polyethylene tubing (PE-50; Clay Adams). Eight months-old mice expressing hTDP-43^WT^ were infused for 2 weeks (0.25 ​μl/h) with ALS-CSF pool or equal volume of NALS-CSF pool.

### Treatments

Both anti-TDP-43-RRM1 domain (E6) monoclonal antibody (mIgG2A), produced as previously described [25], and the isotype control (mIgG2A) mouse monoclonal antibody (807.33) anti-G1 protein of La Crosse virus were purchased from MédiMabs after purification and lyophilization of the antibody from media of hybridoma cells. Lyophilized antibodies were resuspended in sterile miliQ water and kept at −20 ​°C until use.

To assess the diffusion of E6 in the brain, the antibody was labeled with Alexa-488 fluorochrome using Alexa fluor Labeling kit (Invitrogen) as indicated in the manufacturer's manual, purified using the Slide-A-Lyzer MINI Dialysis Unit from ThermoFisher and kept at −20 ​°C until use. One year old hTDP-43^WT^ mice were injected i.c.v. (1.50 ​mm lateral, −1.00 ​mm antero-posterior from Bregma) with 10 ​μl of 1 ​mg/ml labeled E6 antibody or PBS at a rate of 1 ​μl/min using a Hamilton syringe. After 24h, mice were sacrificed as described above to collect the tissues for visualisation of the antibody distribution.

I.t. administration of the treatment started 10 days before the CSF-containing pumps implantation. Mice were injected twice a week with 20 ​μL volume of PBS or 25 ​μg (1.25 ​μg/μL) purified monoclonal antibody, for a total of 7 injections (Supplemental Fig. 2a). The solution was slowly injected into the dura (L4–L5 intervertebral space) using a sterile Hamilton syringe. The presence of a reflex contraction of the tail was considered indicative of a successful administration. The syringe was removed 30 ​s after the end of the injection to minimize CSF and solution leakage. For i.c.v. administration of the treatment, a total of 5 ​ ​μl of PBS, or 25 ​μg (5 ​μg/μl) of purified monoclonal antibody solution, was added to ALS-CSF pool in the pump before implantation (Supplemental Fig. 3b).

### Behavioral analyses

To evaluate the gait pattern, gait analysis was done based on a previously reported method [26]. To assess the memory impairment, mice were subjected to novel object recognition test (NOR). NOR was done as a 3-day test, as previously described [27], in a 20 ​× ​50 ​× ​30 ​cm Plexiglas box for 5 ​min per session. Passive avoidance (PA) test was conducted as described earlier [28] with minor modifications. The mice were first conditioned in a light-dark chamber. On the second day, mice received foot-electric shock of 0.5 ​mA for 4 ​s upon entry in the dark chamber. On the third day, the latency time to enter the dark compartment was measured, a cut-off time of 300 ​s was applied.

### Tissues collection

Following 14 days of i.c.v. infusion, tissues were collected after deep anesthesia with 10 ​μl/g of pentobarbital (12 ​mg/ml, CERVO Brain Research Center animal facility). For protein analyses, male mice were perfused intracardially with cold 0.1 ​M PB. Brain and spinal cord tissues were rapidly collected, frozen in liquid nitrogen, and stored at −80 ​°C until used for protein extraction. To reduce confounding effects due to the surgery or the presence of the pump, we used the brain cortex lysate from the contralateral side of the pump implantation for protein levels analysis. For histological analyses, female mice were first perfused intracardially with 0.1 ​M cold PB and were subsequently perfused with cold 4 ​% paraformaldehyde (PFA). Tissues were then collected, postfixed for 24h in 4 ​% PFA, and cryoprotected in sucrose 30 ​%. Spinal cord and brain tissues were then cut in 25 ​μm thick sections with a Leica frozen microtome and stored in a cryoprotective solution at − 20 ​°C until further use. The muscle tissues from perfused mice were cut in 100 ​μm thick cryosections on a cryostat and stored directly at − 20 ​°C.

### Cell culture

*In vitro* experiments were carried out on NSC-34 ​cells expressing the human TDP-43^WT^ [15,[29], [30], [31], [32]] and BV2 cells [33], cultured in a humidified atmosphere of 95 ​% air/5 ​% CO_2_ in a 37 ​°C incubator in DMEM supplemented with 10 ​% FBS and 1 ​% sodium pyruvate. NSC-43-hTDP-43^WT^ cells were seeded at a density of 5X10^4^ ​cells/ml, whereas BV2 cells at a density of 3X10^4^ ​cells/ml. Cells were allowed to grow until 70–80 ​% confluence was reached. The medium was changed with serum free medium 2h prior CSF treatment. Cultures were then exposed to 10 ​% v/v Dulbecco's PBS (DPBS), NALS-CSF or ALS-CSF pools in DMEM for 48h. During the last 24h, cells were treated with E6 antibody (10 ​μg/ml), control antibody or equal volume of PBS (Supplemental Fig. 2a). The medium of BV2 cells was collected and kept at −20 ​°C until use. Cells were washed once with DPBS, and then lysed.

To study the effect of E6 antibody on NF-κB activation of BV2 cells exposed to ALS-CSF, cells were first treated with E6 antibody (2.5 ​μg/ml), control antibody or PBS for 3h. Then, CSF (10%v/v) was added for an additional 6h (Supplemental Fig. 2c) before lysis.

### Luciferase assay

Luciferase activity was performed as previously described [34], with minor modifications. Briefly, NSC-34-luc-p65 [15] or BV2-luc-p65 [34] cells were seeded in 48 well plates. After exposure to CSF and antibody treatment described above, cells were lysed with 100 ​μl of glo-lysis buffer (Promega Corp., Madison, USA). Lysates were added to a 96 well plates in duplicates for each sample. An equal volume of Bright Glo luciferase assay system (Promega Corp., Madison, USA) was added to each well and the luciferase activity (RLU, relative luminescence units) was measured using an automated plate reader (Enspire, Perkin Elmers Waltham, USA). The luminescence was then normalized to the total protein for each sample, to reduce variability due to cell proliferation/death, and the changes were plotted as fold changes of the control (PBS or NALS-CSF). The pro-inflammatory factor LPS was used as positive control for BV2-luc-p65 ​cells after an exposition to 500 ​ng/ml for 3h.

### Cytokine array

The inflammatory cytokines secretion profile was analyzed with a mouse cytokine antibody array (RayBio Mouse Inflammation Antibody Array 1; RayBiotech) as previously described, with minor modifications [35]. Briefly, for each condition, medium of BV2 cells exposed to CSF and antibody treatment (n ​= ​6 biological replicates per condition) were pooled and incubated with the array membrane overnight at 4 ​°C. After washes, membranes were incubated with biotin-conjugated antibodies overnight at 4 ​°C and then with HRP-Streptavidin for 2h at room temperature. Chemiluminescence was aquired with a ChemiDoc MP Imaging System (Bio-Rad). Images were analyzed using the ImageJ software to measure the optical density of each spot on the membrane. Detection and quantification were performed according to RayBiotech protocols.

### Immunofluorescence, image acquisition and analysis

For the immunofluorescence studies, the spinal cord and brain sections were subjected to antigen retrieval in 0.01 ​M citrate buffer (pH 6.0), equilibrated with PBS (pH 7.4), washed thrice with PBS-0,25%Triton-x (PBS-T), and blocked with 5 ​% BSA or 10 ​% normal goat serum in PBS-T. The sections were then incubated overnight with the primary antibodies of interest (Table 2) in 3 ​% BSA or 1 ​% NGS in PBS-T, followed by appropriate fluorophore-tagged secondary antibodies for 2h at RT. The sections were thoroughly washed with PBS-T and incubated with Hoechst 1:1000 in PBS for 2 ​min. Sections were finally mounted and visualized under a confocal laser scanning microscope (Nikon Instruments, Japan) using a low magnification objective (25x). Analyses were conducted on maximum intensity projection of z-stacks. At least 3 sections per mouse were considered for the analysis.

**Table 2:** List of the antibodies used in the study.

Antibody	Species	Company	#Cat.	Dilution		Temp	Hrs
				IF	WB		
Anti-Arginase-1	Goat polyclonal	SCBT	SC-18354	–	1:1000	4 ​°C	16
Anti-ChAT	Goat polyclonal	Millipore	AB144P	1:250	–	4 ​°C	16
Anti-GAPDH	Mouse monoclonal	SCBT	SC-32233	–	1:2000	4 ​°C	16
Anti-human TDP-43	Mouse monoclonal	Abnova	H00023435	–	1:1000	4 ​°C	16
Anti-Iba1	Rabbit polyclonal	Wako	019–19741	1:500	1:1000	RT/4 ​°C	16
Anti-iNOS	Rabbit monoclonal	Abcam	Ab178945	–	1:1000	4 ​°C	16
Anti-Macrosialin/CD68	Mouse monoclonal	Millipore	MA1435	–	1:1000	4 ​°C	16
Anti-NeuN	Mouse monoclonal	Millipore	MAB377	1:500	1:1000	RT/4 ​°C	16
Anti-NeuN	Rabbit monoclonal	Cell signaling	12943 (D3S31)	1:500	–	RT	16
Anti-NfL	Mouse monoclonal	Sigma	N5139 (NR4)	1:500	1:1000	RT/4 ​°C	16
Anti-NfH	Mouse monoclonal	BioLegend	801701	1:250	–	RT	16
Anti-P84	Mouse monoclonal	Abcam	AB487	–	1:1000	4 ​°C	16
Anti-phospho-p65	Mouse monoclonal	Cell signaling	3036S	–	1:1000	4 ​°C	16
Anti-SV2	Mouse monoclonal	DSHB	SV2	1:100	–	RT	16
Anti-TDP-43	Rabbit polyclonal	Proteintech	12892-1-AP	1:500	1:1000	RT/4 ​°C	16
Anti-goat-HRP	Donkey polyclonal	Invitrogen	A15999	–	1:2000	RT	2
Anti-mouse IgG2A-HRP	Goat polyclonal	Invitrogen	A-10685	–	1:1000	RT	2
Anti-mouse IgG-Alexa 488	Goat polyclonal	Invitrogen	A-11001	1:500	1:2000	RT	2
Anti-mouse IgG-Alexa 555	Goat polyclonal	Invitrogen	A-21424	1:500	1:2000	RT	2
Anti-mouse IgG-Alexa 647	Goat polyclonal	Invitrogen	A-21235	1:500	1:2000	RT	2
Anti-mouse-HRP	Goat polyclonal	Invitrogen	31430	–	1:2000	RT	2
Anti-rabbit IgG-Alexa 488	Goat polyclonal	Invitrogen	A-11008	1:500	1:2000	RT	2
Anti-rabbit IgG-Alexa 555	Goat polyclonal	Invitrogen	A-21428	1:500	1:2000	RT	2
Anti-rabbit IgG-Alexa 647	Goat polyclonal	Invitrogen	A-21244	1:500	1:2000	RT	2
Anti-rabbit-HRP	Goat polyclonal	Invitrogen	31460	–	1:2000	RT	2
Bungarotoxin-Rhodamine	–	Invitrogen	T1175	1:500	–	RT	0,5

IF, Immunofluorescence; WB, Western blot; Temp, Temperature; RT, Room temperature.

The nucleus/cytoplasmic ratio of TDP-43 was determined for each NeuN ​+ ​motor neuron-shaped large cells (area ≥250 ​μm^2^) of the ventral horns of the lumbar spinal cord with visible nucleoli or in the brain motor cortex for each NeuN ​+ ​cell (area ≥100 ​μm^2^). Total integrated density (ID) of TDP-43 for individual neurons was measured using freehand selection feature of ImageJ to mark cell soma/nucleus as the regions of interests (ROIs). The nuclear TDP-43 ID for each neuron was determined by analyzing the intensity corresponding to the nuclear ROI based on the blue (DAPI) channel for each neuron. Cytoplasmic TDP-43 ID was determined by subtracting the nuclear TDP-43 ID from the ID of the entire soma and the nuclear to cytoplasmic intensity ratio (N/C ratio) was averaged for each mouse. At least three slices per mouse were analyzed.

Tibialis muscles were prepared as previously described [36] for NMJ denervation analysis. Briefly, a combination of anti-synaptic vesicle 2 (SV2) and anti-NfH was used to stain the nerve terminal for the presynaptic component, whereas bungarotoxin (Btx), binding to acetylcholine receptors, was used to stain the postsynaptic component of the NMJ on the muscle. The sections were then mounted on slides and images acquired by confocal microscope. Analysis was carried out by counting the number of all fully innervated NMJs, as well as fully or partially denervated NMJs. Between 39 and 216 NMJs were analyzed per mouse, for at least 3 mice per condition.

For microgliosis assessments, an automated intensity detection threshold (Fiji) was applied to images acquired at low magnification (25 ​× ​), and elements bigger than 35 ​μm were selected and analyzed for area distribution [25].

Neuronal loss was assessed in spinal cord ventral horns by counting the NeuN ​+ ​ChAT ​+ ​cells ≥250 ​μm^2^ in each frame corresponding to a ventral horn.

### Statistical analysis

Statistical analyses were performed using PRISM software versions 8.0.2 to 10.2.0 for Windows (GraphPad). Paired and unpaired 1-tailed *t*-test, 1- or 2-way ANOVA followed by post hoc tests for multiple-comparison correction or repeated-measures correction were performed according to the experimental design. Normal distribution and the homoscedasticity of data were verified using Shapiro–Wilk's test. Differences were considered significant when *p* ​< ​0.05. No power analysis was performed before animal treatment, but sample size is consistent with other studies in the field [15,25,37]. Biochemical and histological experiments were always performed using at least 4 individual mice for each experiment and three technical replicates (averaged).

## Results

### ALS-CSF contains more TDP-43 and induces p65-NFκB activation *in vitro*

**Fig. 1:**
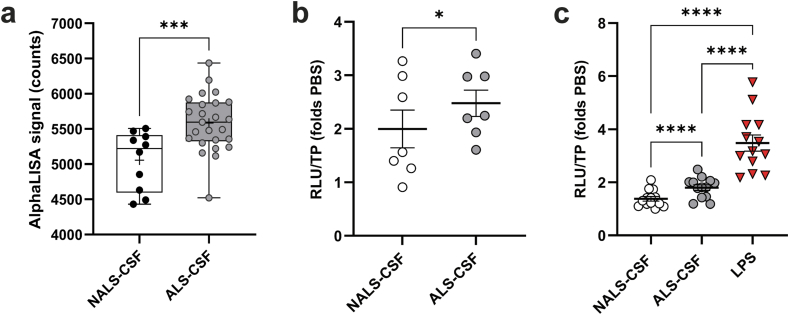
sALS-CSF samples contained elevated levels of TDP-43 species and induced NFκB activation in NSC-43-luc-p65 and BV2-luc-p65 ​cells. **(a)** Levels of TDP-43 in sALS-CSF (5583 ​± ​80.91; n ​= ​25) and control NALS-CSF (5056 ​± ​131.5; n ​= ​10) samples were measured with AlphaLISA. Data are represented as mean ​± ​SEM of the AlphaLISA counts detected, unpaired one-tailed *t*-test analysis (p ​= ​0.0008). **(b**–**c)** NFκB activation was evaluated in reporter cell lines **(b)** neuronal NSC-34-luc-p65 (n ​= ​7 biological replicates; NALS-CSF ​= ​1.998 ​± ​0.352, ALS-CSF ​= ​2.479 ​± ​0.246; p ​= ​0.0161) and **(c)** microglial BV2-luc-p65 (n ​= ​13 biological replicates; NALS-CSF ​= ​1.378 ​± ​0.087, ALS-CSF ​= ​1.798 ​± ​0.109, LPS ​= ​3.481 ​± ​0.304; p ​< ​0.0001) by measuring the luciferase activity (RLU, Relative Luminescence units) after 48h exposition to PBS, CSF (10%v/v) or 3h exposition to pro-inflammatory factor LPS (500 ​ng/ml). Total loaded proteins (TP) were used to normalize the signal. Data are expressed as mean ​± ​SEM of PBS fold change. Paired one-tailed *t*-test or One-way ANOVA followed by Tukey's multiple comparisons test. ∗p ​≤ ​0.05, ∗∗∗p ​≤ ​0.001, ∗∗∗∗p ​≤ ​0.0001.

Based on the hypothesis that CSF transmits the pathology by carrying toxic factors such as TDP-43 [22], we measured the levels of TDP-43 by AlphaLISA in individual CSF samples. The results revealed that the TDP-43 levels were significantly higher in ALS-CSF than control NALS-CSF (Fig. 1a). While ApoB was recently reported as a major toxic factor of ALS-CSF [20], the ALS-CSF samples used in our study did not show increased levels of ApoB compared to control NALS-CSF (Supplemental Fig. 1a).

As variability in levels of toxic factors may occur in different ALS-CSF samples, due to the heterogeneity of the pathology, we decided to use pools of CSF samples from sporadic ALS patients (ALS-CSF, n ​= ​25) and from non-ALS (NALS) controls (NALS-CSF, n ​= ​10) for our studies. Each CSF pool was then tested for its capacity to induce pathological changes, such as p65-NFκB activation [33,38]. Previous studies reported that exposition of neuronal cells to ALS-CSF induced NFκB activation [15]. We therefore treated NSC-34-p65-luc motor neuron-like cells [15] with 10 ​% v/v of pooled CSF for 48h (Supplemental fig. 2a i) and measured luciferase activity as a reporter of NFκB activation. Same experiments were performed with BV2-p65-luc microglial cells [34] to investigate the ability of ALS-CSF to induce NFκB activation in microglia [38]. Our results revealed that exposure to ALS-CSF induced a significant increase in NFκB activation in both cellular types when compared to NALS-CSF (Fig. 1b–c). A 3h treatment with LPS was used as a control of NFκB activation in BV2-p65-luc cells (Fig. 1c).

### E6 antibody is internalized in cultured cells exposed to ALS-CSF

**Fig. 2:**
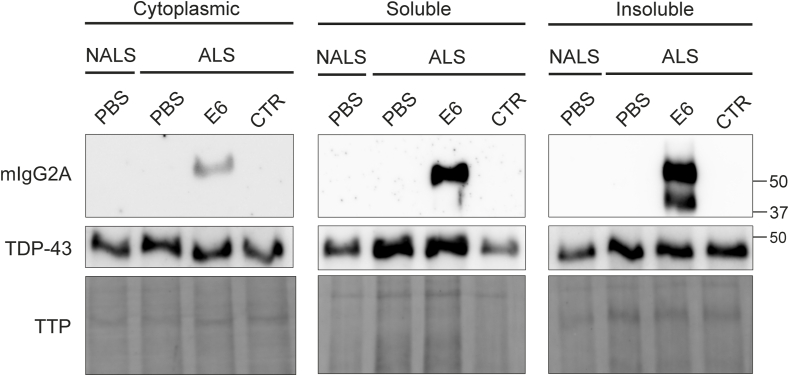
E6 antibody penetrated NSC-34-hTDP-43^WT^ cells exposed to ALS-CSF. Representative immunoblots after SDS-PAGE of mIgG2A and TDP-43 in cytoplasmic, soluble and insoluble protein fractions from NSC-34-hTDP-43^WT^ cells exposed 48h to sALS-CSF or control NALS-CSF and treated for the last 24h with E6 antibody or control antibody (CTR, 10 ​μg/ml), or equal volume of PBS. Supplemental Fig. 2c confirms that the fractioning was successful. Total transferred proteins (TTP) were used as loading reference.

Before evaluating the therapeutic potential of E6 antibody in our CSF-induced ALS model, we investigated, *in vitro,* its ability to penetrate cells exposed to ALS-CSF. We exposed NSC-34-hTDP-43^WT^ and BV2 cells to ALS-CSF or control NALS-CSF and treated them with 10 ​μg/ml of E6 antibody or isotype control antibody, or equal volume of PBS for 24h (Supplemental fig. 2a i). Using an anti-mIgG2A, both E6 and control antibody were detected in the total lysates of BV2 cells (Supplemental Fig. 2b). Analysis of NSC-34-hTDP-43^WT^ cells lysates also revealed the presence of E6 antibody, but not of the control antibody (Fig. 2). These results are consistent with those of Pozzi et al. (2020) suggesting a better retention of E6 antibody when compared to a control antibody [37]. This is likely due to antibody interaction with the cytoplasmic TDP-43 target (Fig. 2) [39]. Indeed, as expected, the E6 antibody was detected in the cytoplasmic fraction of NSC-34-hTDP-43^WT^ cells as well as in the insoluble fraction where pathological TDP-43 can be found (Fig. 2) [15]. No decrease in cell survival was observed, confirming the absence of toxicity of E6 antibody (Supplemental Fig. 2d). To investigate the interaction between E6 antibody and TDP-43, ALS-CSF samples were incubated with magnetic beads coupled with E6 antibody, the control antibody or a commercial antibody against TDP-43. The precipitation of the antibody-magnetic bead complexes resulted in a reduction of CSF-TDP-43 levels by 12 ​% after incubation with E6 antibody (p ​= ​0.0291) compared to the control antibody, confirming the target engagement between E6 antibody and TDP-43 (Supplemental Fig. 2e).

### I.t. delivery of E6 antibody improved motor performance and reduced spinal TDP-43 proteinopathy in mice infused with ALS-CSF

To induce ALS-like pathology [15], pooled ALS-CSF (100 ​μl) were infused i.c.v. in hTDP-43^WT^ mice (8 months of age) for two weeks using a mini-osmotic pump. As control, NALS-CSF samples were also infused in another group. The rationale for using mice expressing human TDP-43 is that, in prion-like propagation of disease, there is potential species to species restriction. Indeed, expression of human TDP-43 in mice was a requirement to trigger TDP-43 proteinopathy by infusion of ALS-CSF [15]. Our group reported previously that ALS-CSF did not trigger TDP-43 pathology in WT mice [15]. Moreover, it should be noted that overexpression of human TDP-43 in the mouse model is mild (only two-folds) because there is a concomitant reduction of 50 ​% in the endogenous mouse TDP-43 levels due to autoregulation of TDP-43 mRNA levels [24,40]. The hTDP-43^WT^ transgenic mice do not develop behavioral or pathological changes during aging [24]. Exposure to ALS-CSF is needed to trigger behavioral deficits and pathological changes [15].

The therapeutic effect of E6 antibody on ALS–CSF–induced pathology was first tested by intrathecal (i.t.) delivery, an approach previously shown to result in spinal motor neuron penetration of antibody [37]. The i.t. treatment with E6 antibody was initiated ten days before starting the i.c.v. infusion of ALS-CSF. The hTDP-43^WT^ mice were injected with 25 ​μg (20 ​μl) of E6 antibody or equal volume of PBS twice a week, for a total of 7 injections (Supplemental Fig. 3a). No adverse effects, such as weight loss or premature death occurred during the study (data not shown), confirming the absence of toxicity of E6 antibody [25,37].

**Fig. 3:**
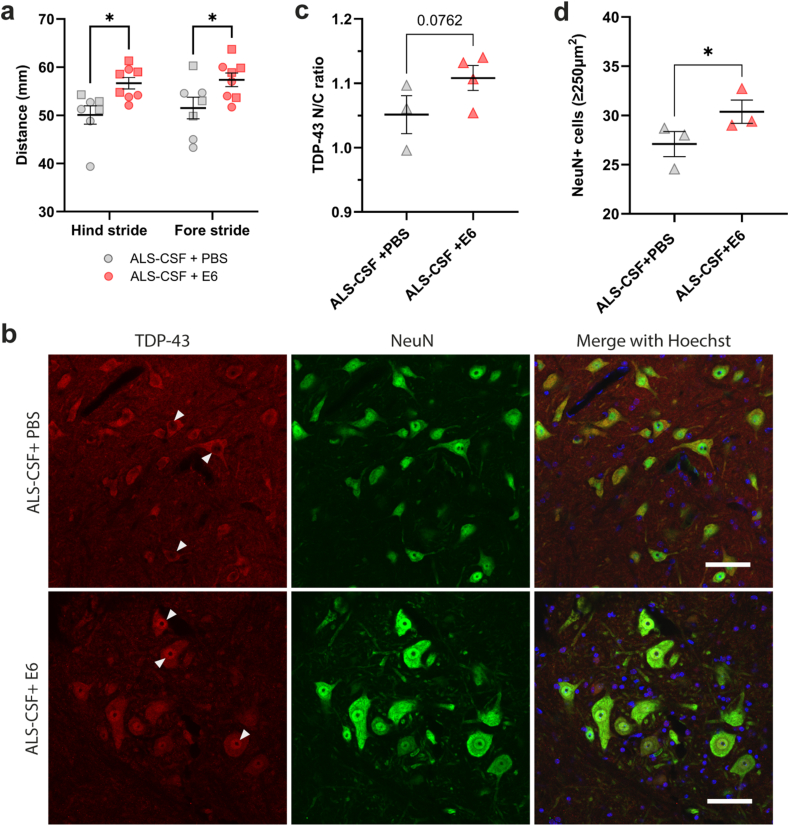
I.t. delivery of E6 antibody improved motor performance and reduced TDP-43 proteinopathy in the lumbar spinal cord caused by infusion of ALS-CSF. **(a)** Gait analysis at day 14 post pump implantation evaluating the stride length (in millimeters) of hind limbs (PBS ​= ​50.07, E6 ​= ​56.66; p-value ​= ​0.0207) and fore limbs (PBS ​= ​51.53, E6 ​= ​57.35; p-value ​= ​0.0431) of ALS-CSF infused mice (n ​= ​8 mice per condition; circle ​= ​male, square ​= ​female) treated i.t. with E6 antibody or equal volume of PBS. Two-way ANOVA followed by Tukey's multiple comparisons test. **(b)** Representative single-channel and merged images of TDP-43 localization in the lumbar spinal cord ventral horn neurons using rabbit polyclonal antibody against TDP-43 ​C-Terminal (TDP-43, red; NeuN, green; Hoechst, blue). Arrowheads highlight cells showing depleted nuclear signal of TDP-43 after ALS-CSF infusion and restored nuclear TDP-43 signal after E6 treatment. Scale bar: 50 ​μm. **(c)** Quantification of nuclear to cytoplasmic ratio of TDP-43 signal in ≥250 ​μm^2^ NeuN ​+ ​neurons (n ​= ​3–4 mice per condition). Data are represented as mean ​± ​SEM (PBS ​= ​1.051 ​± ​0.0295, E6 ​= ​1.108 ​± ​0.0194; p ​= ​0.0762). Unpaired one-tailed *t*-test. **(d)** Quantification of ≥250 ​μm^2^ NeuN ​+ ​cells per hemisection (ventral horn) of the lumbar spinal cord (n ​= ​3 mice per condition). Data are represented as mean ​± ​SEM (PBS ​= ​27.09 ​± ​1.177, E6 ​= ​30.37 ​± ​1.281; p-value ​= ​0.0373). Paired one-tailed *t*-test. ∗p ​≤ ​0.05.

The mouse motor performance was analyzed during the two weeks of ALS-CSF infusion by gait analysis. Results revealed an increased stride length of both hind- and fore-limbs for E6-treated mice as compared to PBS-treated mice (Fig. 3a), suggesting that E6 antibody treatment reduced motor deficits induced by ALS-CSF infusion.

Spinal cord samples were analyzed for the presence of E6 antibody by western blotting. In samples from E6-treated mice, an anti-mIgG2A revealed a band at 50 ​kDa corresponding to the heavy chain of antibody (Supplemental Fig. 3b). This band was absent in samples from the PBS-treated mice. Immunofluorescence of the lumbar spinal cord revealed cytoplasmic mislocalization of TDP-43 in ventral horn neurons of mice infused with ALS-CSF and injected with PBS (Fig. 3b).

In contrast, E6-antibody treatment of ALS-CSF infused mice resulted in high TDP-43 nuclear signal with low cytoplasmic TDP-43 signal (Fig. 3b). The ratios of nuclear to cytoplasmic staining for TDP-43 have been quantified in ≥250 ​mm^2^ neurons, corresponding to motor neurons [[41], [42], [43], [44]]. The quantification revealed a tendency of increased nuclear to cytoplasmic ratio of TDP-43 in E6-treated mice compared to PBS-treated mice (Fig. 3c). Furthermore, i.t. injection of E6 antibody protected spinal neurons from the toxicity of ALS-CSF infusion. NeuN ​+ ​cells larger than 250 ​μm^2^, were counted in ventral horns and results revealed that E6-treated mice exhibited 12 ​% more neurons than PBS-treated mice (Fig. 3d). Thus, i.t. delivery of E6 antibody protected spinal neurons from degeneration induced by ALS-CSF infusion in hTDP-43^WT^ mice.

### I.c.v. delivery of E6 antibody improved motor and cognitive performances of hTDP-43^WT^ mice infused with ALS-CSF

I.t. injection into the spinal fluid is a method that resulted in the detection of E6 antibodies in spinal neurons but not in brain neurons [37]. However, i.c.v. injection led to the detection of the E6 antibody in the cortex and the hippocampus of TDP-43^A315T^ mice [37]. So, to further evaluate the therapeutic efficacy of our anti-TDP-43 antibody in ALS–CSF–induced pathology in the brain, we decided to test the effects of E6 antibody when co-infused i.c.v. with ALS-CSF in the hTDP-43^WT^ mice.

**Fig. 4:**
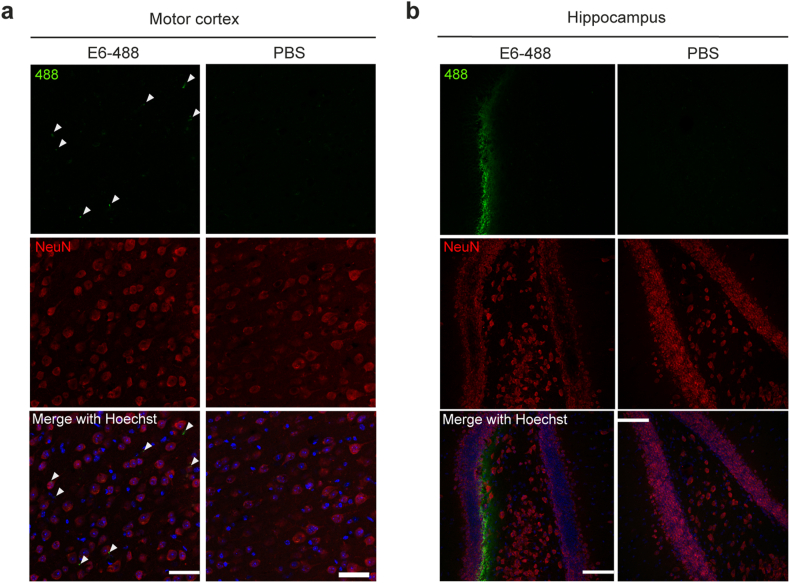
E6 antibody diffused in the brain motor cortex and hippocampus after i.c.v. delivery. Representative single-channel and merged images showing the presence of E6 antibody in **(a)** the brain motor cortex (arrowheads) and **(b)** the hippocampus of mice 24h post-i.c.v. injection of 488-conjugated-E6 (10 ​μg) or equal volume of PBS. (E6 antibody, green; NeuN, red; Hoechst, blue). Scale bar: motor cortex ​= ​50 ​μm, hippocampus ​= ​100 ​μm.

To confirm the diffusion of the antibody in brain tissues after i.c.v. delivery in hTDP-43^WT^ mice infused with ALS-CSF, we carried out an experiment with the E6 antibody labeled with 488 fluorophore. At 24h after a single i.c.v. injection of E6-488 antibody (10 ​μg) or equal volume of PBS, mice were sacrificed, and brain tissues were analyzed. Confocal microscopy revealed the presence of E6-488 antibody in the motor cortex (Fig. 4a) as well as in the hippocampus (Fig. 4b) confirming previous observations of antibody distribution after i.c.v. injection [37].

To test the capacity of the E6 antibody to recognize TDP-43 during two-weeks treatment, we evaluated its ability to detect recombinant human TDP-43 (rhTDP-43) after incubation at 37 ​°C. After 7 or 14 days at 37 ​°C, the different samples of antibody were incubated on a direct ELISA plate coated with rhTDP-43 and then detected using an HRP-conjugated anti-mIgG2A. Over time, the E6 antibody capacity to recognize rhTDP-43 decreased when compared to fresh E6 antibody. Nonetheless, after 14 days at 37 ​°C, E6 antibody was still able to recognize rhTDP-43 with signal intensity higher than the fresh control antibody (Supplemental Fig. 4a). These results suggest that the E6 antibody should remain functional during the 14-day period of infusion into the mouse CSF.

Therefore, to assess the therapeutic effects of E6 antibody, we administered the antibody by i.c.v. co-infusion with ALS-CSF in hTDP-43^WT^ mice. This was simply achieved by adding 25 ​μg of E6 antibody (5 ​ ​μl) in the pump containing the ALS-CSF (95 ​μl) for i.c.v. infusion. The same approach was used to deliver the control antibody or PBS (Supplemental Fig. 4b).

**Fig. 5:**
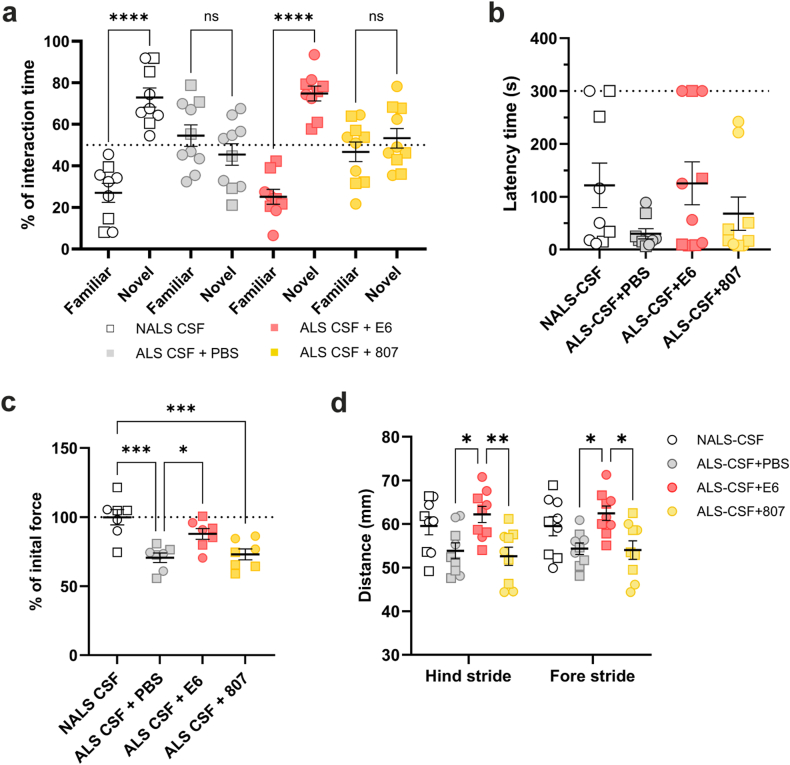
I.c.v. delivery of E6 antibody improved motor and cognitive performance. **(a)** Novel Object Recognition test after 12 days of CSF and antibody infusion (n ​= ​9–10 mice per condition). The graph shows the percentage of interaction time spent with each object. One-way ANOVA followed by Šidák's multiple comparisons test. Dotted line represents 50 ​% of interaction time. **(b)** Passive avoidance test analysis at 14 days of CSF and antibody infusion showing latency time in seconds before moving to the dark room (n ​= ​9 mice per condition; NALS-CSF ​= ​121.7 ​± ​42.12, ALS-CSF ​+ ​PBS ​= ​29.91 ​± ​9.596, ALS-CSF ​+ ​E6 ​= ​125.4 ​± ​40.83, ALS-CSF ​+ ​CTR Ab ​= ​68.01 ​± ​31.40). Kruskal-Wallis test. Dotted line represents cut-off time. **(c)** Grip strength analysis showing the muscle force after 14 days of CSF infusion (n ​= ​7 mice per condition). Data are shown as percentage of their individual force before pump implantation (initial force) (NALS-CSF ​= ​99.87 ​± ​5.53, ALS-CSF ​+ ​PBS ​= ​70.63 ​± ​3.385, ALS-CSF ​+ ​E6 ​= ​87.84 ​± ​3.848, ALS-CSF ​+ ​CTR Ab ​= ​73.01 ​± ​3.941). One-way ANOVA followed by Tukey's multiple comparisons test. Dotted line represents 100 ​% of initial force. **(d)** Gait analysis evaluating stride length in millimeters of hind (NALS-CSF ​= ​59.57 ​± ​2.053, ALS-CSF ​+ ​PBS ​= ​53.856 ​± ​1.821, ALS-CSF ​+ ​E6 ​= ​62.211 ​± ​1.850, ALS-CSF ​+ ​CTR Ab ​= ​52.6 ​± ​2.079) and fore limbs (n ​= ​10 mice per condition; NALS-CSF ​= ​59.511 ​± ​2.199, ALS-CSF ​+ ​PBS ​= ​54.333 ​± ​1.305, ALS-CSF ​+ ​E6 ​= ​62.467 ​± ​1.659, ALS-CSF ​+ ​CTR Ab ​= ​54.011 ​± ​2.131) after 12 days of CSF and antibody infusion. Two-way ANOVA followed by Tukey's multiple comparisons test. Data are represented as mean ​± ​SEM. ∗p ​≤ ​0.05, ∗∗p ​≤ ​0.01, ∗∗∗p ​≤ ​0.001, ∗∗∗∗p ​≤ ​0.0001. (Males ​= ​circle, females ​= ​square). CTR Ab ​= ​control antibody.

As the infusion of ALS-CSF in hTDP-43^WT^ mice resulted in behavioral impairments [15], cognitive and motor performances were evaluated during the treatment (Supplemental Fig. 4b). First, we assessed cognitive performance of i.c.v.-infused mice by evaluating their memory capacity using novel object recognition (NOR) and passive avoidance (PA) tests. When tested by NOR at day 12, the hTDP-43^WT^ mice infused with ALS-CSF along with E6 antibody spent significantly more time exploring the novel object (75 ​%) than the familiar one (25 ​%), meaning that mice remembered the familiar object and had an increased interest in the novel object. In contrast, the hTDP-43^WT^ mice infused with ALS-CSF and PBS or control antibody showed no preference for the novel object (45 ​% and 55 ​% respectively) (Fig. 5a). Total exploration time did not vary between conditions (Supplemental Fig. 4c). The PA test, performed at day 14, showed that the latency to go to the dark room was increased by ∼3 folds in E6-treated mice when compared to PBS-treated mice (Fig. 5b). It is noteworthy that, after the electric shock received 24h before in the dark room, all hTDP-43^WT^ mice infused with ALS-CSF and PBS moved to the dark room quickly, meaning that mice forgot the adverse event. In contrast, a third of hTDP-43^WT^ mice infused with ALS-CSF and E6 antibody spent the whole duration of the test in the light room (Fig. 5b). Thus, the results obtained from NOR and PA tests suggest that E6 antibody reduces the memory deficits induced by the ALS-CSF infusion.

The motor performance testing has been limited to the grip strength measurement and gait analysis. The behavioral results were consistent with the extent of neuromuscular denervation determined subsequently for the tibialis muscle from the different animal groups. At 14 days post pump-implantation, the hTDP-43^WT^ mice infused with ALS-CSF plus E6 antibody maintained a greater percentage of their initial force (determined before pump implantation) compared to PBS- or control antibody-treated mice (Fig. 5c). The muscle force of E6-treated mice revealed to be 17 ​% superior to PBS-treated mice, and 15 ​% superior to control antibody-treated mice. This suggests that E6 treatment had a protective effect against muscle strength decline induced by ALS-CSF infusion. An improvement of motor deficits was also revealed by the gait analysis. Indeed, after 12 days of infusion, E6-treated mice exhibited a significantly longer stride (15 ​%–18 ​%) compared to PBS- or control antibody-treated mice respectively (Fig. 5d), suggesting that E6 antibody reduced motor deficits induced by the ALS-CSF infusion.

### I.c.v. delivery of E6 antibody reduced TDP-43 proteinopathy in motor cortex of hTDP-43^WT^ mice infused with ALS-CSF

Brain and spinal cord tissue sections from hTDP-43^WT^ mice i.c.v. infused with CSF and antibodies were collected for biochemical analysis and fluorescence microscopy after two weeks of infusion. First, the presence of E6 ntibody in the motor cortex and lumbar spinal cord of E6-treated mice was confirmed. SDS-PAGE of brain extracts followed by western blotting with an anti-mIgG2A antibody revealed a band at ∼50 ​kDa corresponding to the heavy chain of antibody in E6-treated mice and to a lower extent in control antibody-treated mice, but not in the PBS treated mice (Supplemental Fig. 4d). Interestingly, a faint band corresponding to E6 antibody was also detected in lumbar spinal cord extracts of some i.c.v. treated mice suggesting a diffusion of the antibody from the ventricles to the spinal regions (Supplemental Fig. 4e).

**Fig. 6:**
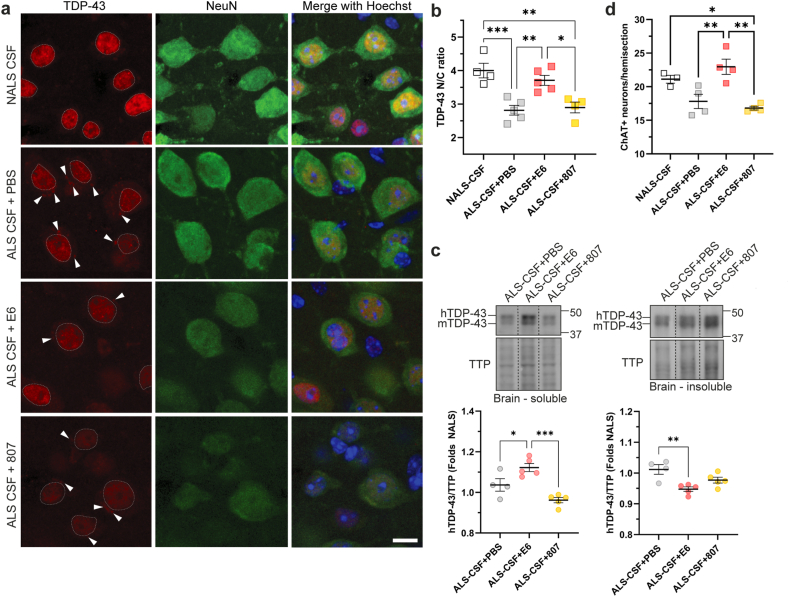
I.c.v. delivery of E6 antibody reduced TDP-43 proteinopathy. **(a)** Representative single-channel and merged images of TDP-43 localization using a rabbit polyclonal antibody against TDP-43 ​C-Terminal on brain (motor cortex) tissues (TDP-43, red; NeuN, green; Hoechst, blue). Dotted line circles the nucleus. Arrowheads point to TDP-43 cytoplasmic inclusions. Scale bar: 10 ​μm. **(b)** Quantification of nuclear to cytoplasmic ratio of TDP-43 signal (n ​= ​4–5 mice per condition; NALS-CSF ​= ​4.003 ​± ​0.216, ALS-CSF ​+ ​PBS ​= ​2.816 ​± ​0.146, ALS-CSF ​+ ​E6 ​= ​3.71 ​± ​0.148, ALS-CSF ​+ ​CTR Ab ​= ​2.9 ​± ​0.162). One-way ANOVA followed by Tukey's multiple comparisons test. **(c)** Representative blots and quantifications of human TDP-43 in soluble and insoluble fractions of brain cortex lysates (n ​= ​4–5 mice per condition). Data are shown as fold of NALS-CSF (Soluble: ALS-CSF ​+ ​PBS ​= ​1.037 ​± ​0.031, ALS-CSF ​+ ​E6 ​= ​1.122 ​± ​0.02, ALS-CSF ​+ ​CTR Ab ​= ​0.962 ​± ​0.013; Insoluble: ALS-CSF ​+ ​PBS ​= ​1.012 ​± ​0.016, ALS-CSF ​+ ​E6 ​= ​0.948 ​± ​0.007, ALS-CSF ​+ ​CTR Ab ​= ​0.977 ​± ​0.01). One-way ANOVA by Tukey's multiple comparison test. Samples were run on the same gel but were noncontiguous. Total transferred proteins (TTP) were used as loading reference. **(d)** Count of ≥250 ​μm^2^ NeuN+ and ChAT ​+ ​cells per hemisection of the ventral horns of the lumbar spinal cord (n ​= ​3–5 mice per condition; NALS-CSF ​= ​21.11 ​± ​0.588, ALS-CSF ​+ ​PBS ​= ​17.81 ​± ​1.056, ALS-CSF ​+ ​E6 ​= ​22.96 ​± ​1.137, ALS-CSF ​+ ​CTR Ab ​= ​16.81 ​± ​0.281). One-way ANOVA followed by Tukey's multiple comparisons test. All data are expressed as mean ​± ​SEM. ∗p ​≤ ​0.05, ∗∗p ​≤ ​0.01, ∗∗∗p ​≤ ​0.001.

Immunofluorescence with an anti-TDP-43 ​C-terminal antibody (Proteintech) confirmed TDP-43 proteinopathy in cortical neurons induced by exposure to ALS-CSF (Fig. 6a) [15]. Indeed, unlike NALS-CSF, i.c.v. infusion of ALS-CSF (supplemented with PBS) provoked a loss of nuclear TDP-43 associated with cytoplasmic mislocalization and aggregation of TDP-43 (Fig. 6a). Remarkably, the treatment with E6 antibody alleviated the TDP-43 proteinopathy triggered by ALS-CSF infusion in cortical neurons of hTDP-43^WT^ mice (Fig. 6a). As observed, the co-infusion of E6 antibody with ALS-CSF led to a reduction of TDP-43 staining in the cytoplasm and increased nuclear signal for TDP-43 when compared to PBS- or control antibody-treated mice. The quantification showed that the ratio of nuclear to cytoplasmic signals of TDP-43 in neurons ≥100 ​μm^2^ increased significantly in E6-treated mice when compared to PBS- and control antibody-treated mice, by 32 ​% and 28 ​% respectively (Fig. 6b).

We further investigated the aggregation of TDP-43 by SDS-PAGE and western blotting of soluble and insoluble fractions from lysates of brain cortex (Fig. 6c). A polyclonal anti-TDP-43 ​C-Terminal antibody revealed two bands corresponding to mouse TDP-43 (mTDP-43, ∼43 ​kDa) and human TDP-43 (hTDP-43, myc-tagged, ∼45 ​kDa) (Fig. 6c). Brain cortex lysates of E6-treated mice exhibited significantly more soluble hTDP-43 than PBS- or control antibody-treated mice and less insoluble hTDP-43 than PBS-treated mice (Fig. 6c). These results suggest that i.c.v. infused E6 antibody mitigated TDP-43 proteinopathy in the brain.

Motor neuron loss in the lumbar spinal cord was determined by counting the number of NeuN ​+ ​ChAT ​+ ​cells bigger than 250 ​μm^2^ in the ventral horns. The quantification revealed that the ventral horns of E6 treated mice exhibit significantly more motoneurons (5–6) than PBS- or control antibody-treated mice (Fig. 6d).

### E6 antibody treatment reduced neuromuscular junction denervation and prevented neurofilament disorganization

**Fig. 7:**
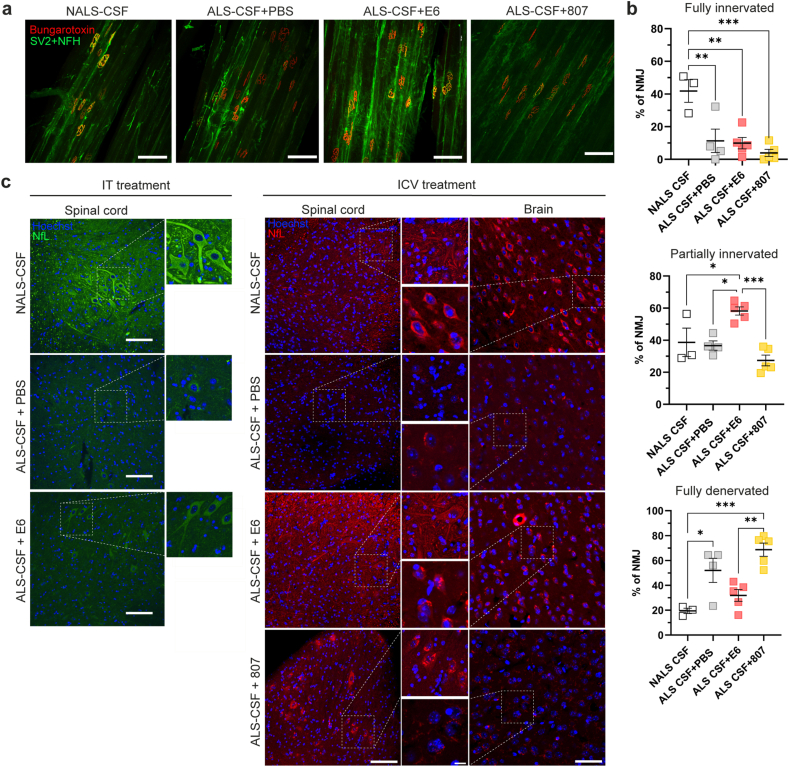
E6 antibody treatment reduced NMJ denervation and prevented Nf cytoskeleton disorganization. **(a)** Representative merged images of neuromuscular junction pre-synaptic (SV2+NfH, green) and post-synaptic (Bungarotoxin, red) components on tibialis muscle tissues of i.c.v. treated mice. Scale bar: 100 ​μm. **(b)** Quantification of NMJ fully innervated (NALS-CSF ​= ​41.87 ​± ​6.93, ALS-CSF ​+ ​PBS ​= ​11.33 ​± ​7.18, ALS-CSF ​+ ​E6 ​= ​9.94 ​± ​3.492, ALS-CSF ​+ ​CTR Ab ​= ​3.94 ​± ​2.15), partially innervated (NALS-CSF ​= ​38.67 ​± ​8.882, ALS-CSF ​+ ​PBS ​= ​36.65 ​± ​2.876, ALS-CSF ​+ ​E6 ​= ​58.16 ​± ​2.553, ALS-CSF ​+ ​CTR Ab ​= ​27.36 ​± ​3.325) and fully denervated (NALS-CSF ​= ​19.50 ​± ​2.155, ALS-CSF ​+ ​PBS ​= ​52.05 ​± ​9.753, ALS-CSF ​+ ​E6 ​= ​31.90 ​± ​4.775, ALS-CSF ​+ ​CTR Ab ​= ​68.68 ​± ​5.352). Data are represented as mean ​± ​SEM, n ​= ​3–5 per condition. One-way ANOVA by Tukey's multiple comparison test, ∗p ​≤ ​0.05, ∗∗p ​≤ ​0.01, ∗∗∗p ​≤ ​0.001. **(c)** Representative merged images of NfL on lumbar spinal cord and brain motor cortex tissues after i.t. (NfL, green; Hoechst, blue) or i.c.v. (NfL, red; Hoechst, blue) treatment with E6 antibody or control antibody (CTR Ab), or equal volume of PBS. Scale bar: 100 ​μm, zoom: 20 ​μm.

To further investigate the therapeutic effects of E6 antibody on motor deficits induced by ALS-CSF infusion, we investigated the extent of neuromuscular junction (NMJ) denervation. Studies have demonstrated that denervation of NMJs is an early event in ALS pathogenesis, occurring before onset of symptoms and pathological changes such as neuronal death [42,45]. Tibialis muscle sections from hTDP-43^WT^ mice infused i.c.v. with CSF and E6 antibody were stained for presynaptic and postsynaptic components of the NMJ (Fig. 7a). For each mouse, all pretzel-like NMJs [36] were counted and classified as fully innervated, partially innervated and fully denervated according to the degree of overlap between pre- and post-synaptic signals. I.c.v. infusion of ALS-CSF significantly decreased the percentage of fully innervated NMJs by ∼30 ​%, regardless of the treatment, when compared to infusion of NALS-CSF (Fig. 7b). However, E6-treated mice infused with ALS-CSF exhibited 22 ​%–31 ​% more partially innervated (58 ​%) NMJs and 20 ​%–37 ​% less fully denervated (32 ​%) NMJs than PBS- (partially innervated: 37 ​%, fully innervated: 52 ​%) or control antibody-treated (partially innervated:27 ​%, fully innervated: 69 ​%) mice (Fig. 7b). These results suggest that E6 treatment had protective effect against NMJ denervation induced by ALS-CSF infusion.

Nf disorganization is another pathological hallmark of ALS and one of the most promising blood biomarker for the disease [[46], [47], [48], [49], [50], [51]]. Mishra et al. [15] showed that ALS-CSF infusion induces a strong disorganization and a reduction in the levels of neuronal intermediate filaments in the spinal cord, including Nf light chain (NfL), that could be linked to selective translational suppression of NfL mRNA due to TDP-43 proteinopathy [52]. Since E6 antibody reduced TDP-43 proteinopathy and promoted the presence of soluble TDP-43 (Fig. 3b–c, Fig. 6a–d), we examined the effect of E6 antibody on NfL pathological changes induced by ALS-CSF. As shown in Fig. 7c, immunofluorescence confirmed that ALS-CSF induced a strong reduction in overall NfL signal and a complete loss of signal in the dendrites of neurons in both the spinal cord and the brain when compared to NALS-CSF infusion (Fig. 7c). E6-treated mice exhibited a partial recovery of NfL distribution with restoration of the NfL signal in dendrites and apparent reduction of NfL inclusions (Fig. 7c). These results demonstrate that E6 treatment attenuated NfL disorganization induced by ALS-CSF infusion.

### E6 antibody promoted beneficial microglial activation

A previous study revealed that E6 antibody was able to reduce NFκB activation *in vitro* in BV2 cells triggered with LPS [37]. Nevertheless, when injected i.t. in TDP-43^A315T^ mice, E6 antibody induced the activation of microglial cells in the spinal cord [37]. We therefore analyzed the degree of activation of microglial cells in our model.

**Fig. 8:**
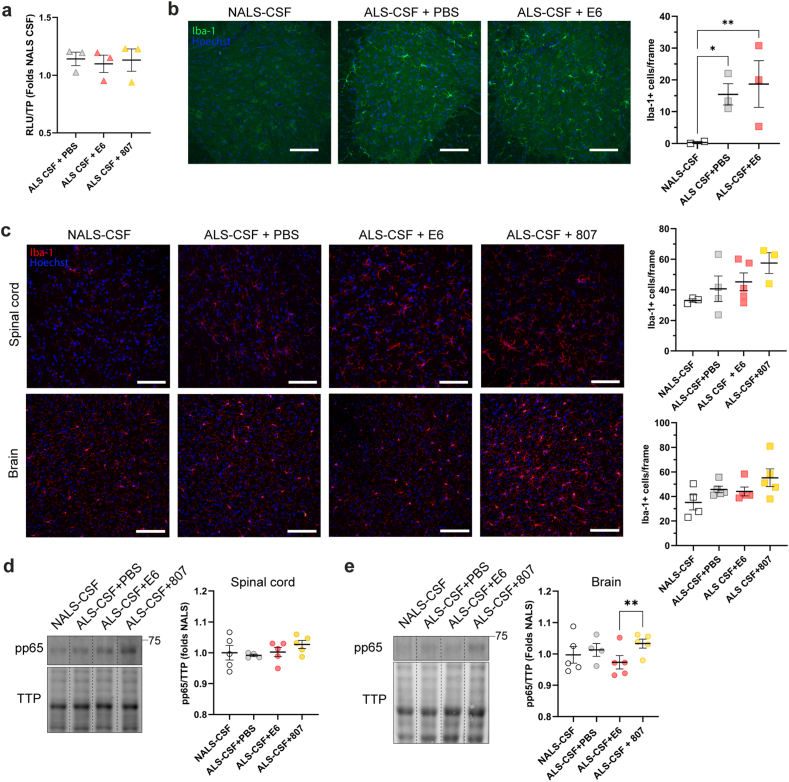
E6 antibody reduced NFκB activation in the brain but did not reduce microgliosis induced by ALS-CSF. **(a)** NFκB activation in BV2-p65-luc (n ​= ​6 biological replicates) by measuring the luciferase activity (RLU, Relative Luminescence Units) after a 9h treatment with 2.5 ​μg/ml of E6 antibody or control antibody, or equal volume of PBS and 6h exposition to CSF. Data are expressed as mean ​± ​SEM of fold change of NALS-CSF. **(b)** Representative merged images and quantifications of Iba-1+ cells per hemisection (Iba-1, Green; Hoechst, Blue) of lumbar spinal cord (ventral horns) of ALS-CSF infused mice (n ​= ​3 mice per condition) after i.t. treatment with E6 antibody or PBS. Scale bar: 100 ​μm. Data are expressed mean ​± ​SEM. **(c)** Representative merged images and quantifications of Iba-1+ cells per hemisection (Iba-1, Red; Hoechst, Blue) of lumbar spinal cord (ventral horns) and brain (motor cortex) tissues of CSF-infused mice (n ​= ​3–5 mice per condition) after i.c.v. treatment with E6 antibody or control antibody, or equal volume of PBS. Scale bar: 100 ​μm. Data are expressed as mean ​± ​SEM. **(d**–**e)** Representative blots and quantifications of pp65 in **(d)** lumbar spinal cord and **(e)** brain cortex lysates of mice (n ​= ​3–5 mice per condition) treated i.c.v. with E6 antibody, PBS or control antibody. Samples were loaded on the same membrane but were not contiguous. Total transferred proteins (TTP) were used as loading reference. Data are expressed as mean ​± ​SEM of NALS-CSF fold change. For all analyses: One way ANOVA by Tukey's multiple comparison test ∗p ​≤ ​0.05, ∗∗, p ​≤ ​0.01. CTR Ab ​= ​control antibody.

As shown in Fig. 8, a pre-treatment with E6 antibody (2.5 ​μg/ml) did not lead to a reduction of NFκB activation in BV2 cells exposed to ALS-CSF for 6h (Supplemental Fig. 2a ii, Fig. 8a). *In vivo*, ALS-CSF infusion in hTDP-43^WT^ mice induced microgliosis in the spinal cord as revealed by the quantification of Iba-1+ cells, but this activation was not inhibited nor exacerbated by i.t. administration of E6 antibody (Fig. 8b). Similarly, i.c.v. administration of E6 antibody did not inhibit nor exacerbate microgliosis in the brain or spinal cord but reduced phosphorylated p65 (pp65) levels in the brain cortex compared to control antibody-treated mice (Fig. 8c–e).

Activated microglia can have either a protective anti-inflammatory and phagocytic phenotype, or a pro-inflammatory one. Moreover, it has been suggested that an increased phagocytic function following activation, notably by immune complexes, can yield to a better clearance of TDP-43, and to a better uptake of the antibody [[53], [54], [55]]. We therefore hypothesized that E6 antibody would increase microglia protective function and investigated the phagocytic capacity of microglia as well as the secretion of pro-inflammatory cytokines induced by E6 antibody after ALS-CSF infusion.

**Fig. 9:**
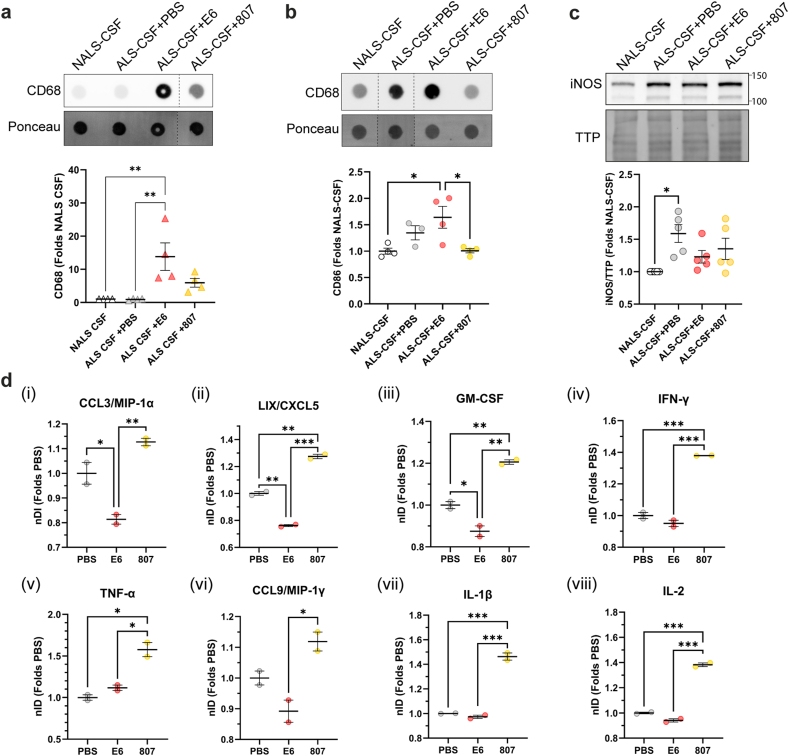
E6 antibody promoted phagocytic capacity and reduced the secretion of pro-inflammatory cytokines by BV2 cells after exposition to ALS-CSF. **(a**–**b)** Representative blot and quantification of phagocytosis marker CD68 in lysates of **(a)** BV2 cells (n ​= ​4 biological replicates, ALS-CSF ​+ ​PBS ​= ​0.9925 ​± ​0.1262, ALS-CSF ​+ ​E6 ​= ​13.81 ​± ​4.167, ALS-CSF ​+ ​CTR Ab ​= ​5.935 ​± ​1.330) exposed 48h to CSF and treated for the last 24h with 5 ​μg/ml of E6 antibody or control antibody, or equal volume of PBS, or **(b)** brain cortex of mice (n ​= ​3–4 mice per condition, ALS-CSF ​+ ​PBS ​= ​1.463 ​± ​0.1504, ALS-CSF ​+ ​E6 ​= ​1.641 ​± ​0.2066, ALS-CSF ​+ ​CTR Ab ​= ​1.006 ​± ​0.0402) infused with CSF and treated i.c.v. with 25 ​μg of E6 antibody or control antibody, or equal volume of PBS. Data are shown as mean ​± ​SEM and are represented as folds of NALS-CSF. Samples were loaded on the same membrane but were not contiguous. Ponceau was used as loading reference. **(c)** Representative blot and quantification of pro-inflammatory marker iNOS in lysates of BV2 cells (n ​= ​5 biological replicates) exposed 48h to CSF and treated 24h with E6 antibody, control antibody or PBS. Data are shown as mean ​± ​SEM and are represented as folds of NALS-CSF (ALS-CSF ​+ ​PBS ​= ​1.59 ​± ​0.1378, ALS-CSF ​+ ​E6 ​= ​1.354 ​± ​0.1640, ALS-CSF ​+ ​CTR Ab ​= ​1.23 ​± ​0.0967). Total transferred proteins (TTP) were used as loading reference. **(d)** Expression analyses of pro-inflammatory cytokines in the medium (pool of 6 biological replicates) of BV2 cells after 48h exposure to ALS-CSF and 24h to E6 antibody, control antibody or PBS. Data are shown as mean ​± ​SEM of normalized Integrated Density (nDI) of each sample shown as folds of PBS. Each sample was run in duplicate for 2 technical replicates. For all analyses: One-way ANOVA by Tukey's multiple comparison test. ∗p ​≤ ​0.05, ∗∗p ​≤ ​0.01, ∗∗∗p ​≤ ​0.001. CTR Ab ​= ​control antibody.

To determine whether E6 antibody can promote a protective phagocytic phenotype of microglia, we investigated the expression levels of the phagocytic marker CD68 in BV2 cells exposed 48h to ALS-CSF and, for the last 24h, to E6 antibody, control antibody or PBS. We performed a dot blot analysis with the lysates loaded onto a membrane and probed with a mouse monoclonal anti-CD68. Results revealed that CD68 levels were increased in E6-treated cells compared to PBS- or control antibody-treated cells (Fig. 9a). The phagocytic capacity of microglia was confirmed by phagocytic assay using fluorescent beads (Supplemental Fig. 5a). Cells were categorized as containing more than 5 beads, containing between 1 and 5 beads and without beads. Quantitative analyses revealed that 39 ​% of the E6-treated cells phagocyted at least one bead, compared to 32 ​% and 33 ​% for PBS- or control antibody-treated cells respectively. Moreover, 16 ​% of E6-treated cells contained more than 5 beads compared to 13 ​% of PBS- or control antibody-treated cells, suggesting a higher phagocytic capacity (Supplemental Fig. 4b). The phagocytic properties of E6-treated microglia were confirmed *in vivo*. Dot blot analysis revealed that the levels of CD68 were increased in brain cortex lysates of E6-treated mice compared to control antibody-treated mice (Fig. 9b). Moreover, pro-inflammatory marker inducible nitric oxide synthase (iNOS) was increased in BV2 cells exposed to ALS-CSF compared to NALS-CSF but tended to decrease in cells treated with E6 antibody when compared to PBS-treated cells (Fig. 9c).

Finally, a cytokine array revealed that the medium of BV2 cells exposed to ALS-CSF and treated with E6 antibody contained less pro-inflammatory cytokines than the cells treated with PBS or control antibody (Fig. 9d). Of note, CCL3/MIP-1α, LIX/CXCL5 and GM-CSF, pro-inflammatory cytokines known to be increased in ALS cases [[56], [57], [58]], were significantly decreased in E6-treated cells compared to PBS- or control antibody-treated cells (Fig. 9d i-iii). It is also noteworthy that levels of pro-inflammatory cytokines IFN-γ, TNF-α, CCL9/MIP-1γ, IL-1β and IL-2 were significantly lower in the medium of E6-treated cells compared to the medium of control antibody-treated cells (Fig. 9d iv-viii). These results suggest that E6 antibody promotes phagocytic function associated with beneficial microglia phenotype and reduces the pro-inflammatory phenotype induced by ALS-CSF exposure.

## Discussion

Here, we report that administration of a full-length antibody, called E6, targeting TDP-43 RRM1 domain attenuated pathological changes and symptoms in a mouse model of sporadic ALS induced by i.c.v. infusion of CSF from sALS patients. In the last fifteen years, various studies have provided evidence of toxicity of ALS-CSF using *in vitro* and *in vivo* paradigms [[11], [12], [13], [14], [15], [16], [17], [18], [19], [20], [21]]. Results presented here further confirm the toxicity of ALS-CSF with transmission of pathogenesis by CSF infusion into mice expressing human TDP-43^WT^.

Increasing efforts have been made on developing antibody-based therapies targeting various proteins involved in ALS [9]. Recently, the use of both single-chain variable fragment (scFv) and monoclonal full-length antibody targeting the RRM1 domain of TDP-43 successfully attenuated TDP-43 proteinopathy in a genetic model of ALS induced by TDP-43^A315T^ mutation [25,37]. Despite these promising results, the efficacy of E6 antibody remained to be proven in context of sporadic cases of ALS, accounting for more than 90 ​% of ALS cases [59]. Here, our results demonstrate the protective effects of the E6 antibody in pathogenic pathways transmitted by the i.c.v. infusion of CSF from sALS in hTDP-43^WT^ mice.

In this study, we demonstrated the protective effects of E6 antibody on behavioral deficits induced by the infusion of CSF from sALS patients [15]. Treatment with E6 antibody led to an amelioration of both motor and cognitive performance (Figs. 3a and 5 a-d). Cognitive performance was not influenced by i.t. treatment with E6 antibody (data not shown), in line with the observation that i.t. injection did not result in detectable diffusion and neuronal penetration of the antibody in the brain [37].

Previous studies demonstrated that the E6 antibody was able to penetrate neurons and microglial cells after i.t. or i.c.v. injection in TDP-43^A315T^ mice, a model of fALS [25,37]. Indeed, studies reported clathrin-mediated endocytosis of antibodies, necessary for the clearance of the target, after the binding of the Fc domain to the Fcγ receptors present on microglia and neuron membranes [39,[60], [61], [62]]. Here, we confirmed the penetration of E6 antibody in neurons and microglial cells exposed to ALS-CSF *in vitro* (Fig. 2a and Supplemental Fig. 2b). Moreover, our results further confirmed that the administration of E6 antibody in the CSF of mice resulted in the diffusion of E6 antibody in the nervous tissues. After i.t. injection, E6 antibody was detected in the lumbar spinal cord (Supplemental Fig. 3b). Moreover, as early as 24h after i.c.v. injection, E6 antibody was detected in the motor cortex (Fig. 4). The E6 antibody was detectable in the brain cortex and spinal cord after 2 weeks of continuous infusion (Supplemental Fig. 4d and e). In contrast to E6 antibody, the isotype control antibody (807.33 anti-G1 protein of La Crosse virus) was not detected in neuron-like cells *in vitro* (Fig. 2a), and to a lesser extent, compared to E6, *in vivo* (Supplemental Fig. 4d). This is likely due to the absence of control-antibody target inside the cells, leading to rapid degradation of the control antibody after its internalization. Increased uptake of control antibody by microglial cells *in vitro* (Supplemental Fig. 2b) can be due to the high expression of Fcγ receptors on the surface of this cellular type and to its enhanced phagocytic function (Fig. 9a and Supplemental Fig. 5) [[63], [64], [65]] in the context of immune activation, neurodegenerative condition and in the presence of immune complexes [[65], [66], [67]].

TDP-43 proteinopathy is a key feature of ALS, occurring in more than 95 ​% of ALS cases, both fALS and sALS [3]. Suppressing the spread of pathological TDP-43 aggregates may represent an important therapeutic strategy for halting the progression of ALS [[6], [7], [8]]. As expected, E6 antibody was detected *in vitro* in the cytoplasmic fraction of neuron-like cells where TDP-43 is mislocalized after an exposure to ALS-CSF (Fig. 2a) [15]. Interestingly, the presence of E6 antibody in the insoluble fraction (Fig. 2a) suggests that E6 binds to aggregated TDP-43, supporting the preference of E6 antibody for the aggregation-prone species of TDP-43 [37,68,69]. Most importantly, *in vivo* treatment with E6 antibody caused the reduction of cytoplasmic accumulation of TDP-43 in motor neurons (Fig. 3b–c, Fig. 6a–b) and of insoluble levels of TDP-43 in the brain motor cortex (Fig. 6c). This is in line with the evidence that internalized E6 antibody promotes TDP-43 degradation by the proteasome through the recruitment, with its Fc domain, of Tripartite motif-containing protein 21 (TRIM21), and possibly by the lysosome machinery [37]. Thus, E6 antibody would reduce the cytoplasmic and insoluble burden of TDP-43 by promoting its clearance via proteasomal degradation. The clearance of cytoplasmic TDP-43 aggregates would restore nuclear translocation of soluble TDP-43 recovery of TDP-43 function.

The attenuation of TDP-43 proteinopathy might provide beneficial effects beyond the clearance of TDP-43 toxic aggregates. The restoration of soluble and nuclear levels of TDP-43 might allow the recovery of important TDP-43 function such as those involved in RNA processing, crucial for many biological processes. Moreover, translation of NfL, a well-known RNA target of TDP-43 [70,71], is altered in the context of TDP-43 proteinopathy, resulting in lower levels of the protein [72]. The i.c.v. infusion of ALS-CSF caused a severe loss NfL signal in dendrites and axons in the spinal cord and brain (Fig. 7c). The ALS-CSF exposure led to formation of NfL aggregates sequestered in neuronal perikaryal, a hallmark of neurodegeneration [15,33]. Remarkably, administration of E6 antibody alleviated the depletion and misdistribution of NfL in spinal and brain neurons (Fig. 7c). Likewise, the E6 treatment contributed to NMJ maintenance. It has been shown that there is a mechanistic link between TDP-43 and NMJ loss [73]. TDP-43 cytoplasmic aggregation can cause defects in the axonal transport of mRNA to the nerve terminal and local translation of peptides in the axonal terminal, contributing to NMJ disruption [74,75]. Moreover, the sequestration of RNA into insoluble complexes may deplete motor neurons of essential proteins for the maintenance of NMJ [73,74]. The increased availability of soluble TDP-43, observed after E6 antibody treatment (Fig. 6c), may contribute to restore downstream pathways implicated in axonal transport or synaptic translation, preventing NMJ denervation (Fig. 7a–b) [74,76,77]. The increased number of spinal motor neurons in E6 treated mice (Figs. 3d and 6d) further confirmed that E6 antibody protects motor neurons from degeneration. Previous cultured cells studies (25, 37) demonstrated that internalization of full length E6 or single chain E6 antibodies did not induce cell death and did not affect nuclear TDP-43 function. Immunofluorescence revealed that E6 antibody localized mainly in the cytoplasm after cell penetration and it was not detected in the nucleus. The E6 antibody is highly specific against TDP-43. The E6, which binds to a small epitope in the RRM1 domain, is unique in that it detects cytoplasmic TDP-43 but not nuclear TDP-43.

Finally, TDP-43 is known to activate NFκB through its interaction with p65 subunit, contributing to the neuroinflammation observed in ALS [33]. While full-length E6 and E6-derived scFv antibodies reduced TDP-43 interaction with p65 and NFκB activation *in vitro* and *in vivo* [25,37], E6 treatment increased microgliosis in brain and lumbar spinal cord of TDP-43^A315T^ mice [37]. These results can be attributed to the immunogenic potential of the Fc domain of full-length antibodies [53,54]. In the context of ALS–CSF–mediated disease, we show that E6 antibody slightly reduced NFκB activation in the brain of ALS–CSF–treated mice (Fig. 8e) without remarkably affecting microgliosis (Fig. 8b–c). This discrepancy could be explained by the fact that NFκB activation was previously demonstrated specifically in neurons, while in this study it has been measured in whole mouse tissues which may mask the effect of E6 on this single cell type.

Microglial activation is a double-edged sword, which can be both anti-inflammatory and neuroprotective [[78], [79], [80]] or pro-inflammatory and neurotoxic [35,78,81,82]. It is widely suggested nowadays that therapies promoting a shift in microglia phenotype from pro-inflammatory to protective are required for treating neurodegenerative diseases [82,83]. Here we show that E6 antibody enhanced microglial phagocytic function and reduced the secretion of pro-inflammatory cytokines (Fig. 9). These microglial changes are most likely the results of both the binding of E6 antibody to Fcγ receptors at the membrane of microglia, activating phagocytosis, and the reduction of TDP-43 burden, known to induce pro-inflammatory activation of microglia [33,84,85]. These observations suggest that, although still activated, microglia acquire an anti-inflammatory and beneficial phenotype after E6 treatment, which contribute to TDP-43 clearance and thus to motor neurons preservation and symptoms attenuation.

What factors contribute to disease transmission via the toxicity of ALS-CSF? Several studies provided insights into factors that could explain, at least partially, ALS-CSF toxicity. In 2019, Tokuda and colleagues reported the increased presence of misfolded SOD1 (mSOD1) in sALS-CSF and that the depletion of mSOD1 attenuated sALS-CSF toxicity toward motor neuron-like cells [86]. A recent study by Wong et al. [20] rather suggest that Apolipoprotein B (ApoB) constitutes the predominant toxic component in the CSF of sALS patients responsible for inducing motor neuron pathology, TDP-43 proteinopathy and death after i.t. injection in mice. However, the levels of ApoB are highly variable between CSF samples. In our study, results showed no increase in ApoB levels in our samples of sALS-CSF compared to NALS-CSF (Supplemental Fig. 1). Accordingly, the development here of TDP-43 proteinopathy in response to sALS-CSF infusion cannot be attributed to CSF ApoB. It has also been reported that ALS-CSF contains more TDP-43 than control individuals CSF [[87], [88], [89], [90], [91], [92]]. Our results further confirm the increased levels of TDP-43 in ALS-CSF (Fig. 1a). Interestingly, a recent study showed that ALS-CSF TDP-43 has a seeding capacity, through prion-like properties, capable of propagating the disease [[6], [7], [8]]. Therefore, a role for TDP-43 species in sALS-CSF toxicity cannot be excluded [[87], [88], [89], [90], [91], [92], [93]]. Further investigations are needed to determine whether E6 antibody can neutralize putative toxic TDP-43 species in the sALS-CSF samples.

Although promising, our results are not without limitations. ALS indeed is a very heterogenous disease in terms of phenotypes, onset, rate of progression, severity and duration. The lack of clinical information on ALS patients who provided the CSF samples used in this study and the number of samples available did not allow us to test subgroups according to clinical features such as sex, age at onset, disease duration, severity and rate of progression. Although few studies have already highlighted possible correlations between clinical features of ALS patients and the toxicity of the CSF [94,95], further research is needed to better understand how CSF samples from patients with distinct clinical features may induce toxicity and impact the disease propagation.

In conclusion, our results suggest that E6 antibody targeting TDP-43 mitigated pathogenic pathways triggered by exposure to CSF of sALS patients. While the therapeutic effects of anti-TDP-43 E6 antibody was previously validated in transgenic mouse models of ALS harboring familial *TARDBP* gene mutations [25,37], we demonstrated here its therapeutic potential in a model of sporadic ALS. Our results suggest that injection into CSF of antibodies targeting TDP-43 should be considered as a new potential therapeutic approach for the treatment of sporadic forms of ALS and of other neurodegenerative disorders with TDP-43 proteinopathy. There are currently clinical trials based on i.t. delivery of antisense oligonucleotide (ASO) targeting specific gene mutations linked to ALS [96]. Similarly, intra-CSF delivery of ‘humanized’ antibody targeting TDP-43 would be a feasible approach applicable to most ALS patients.

## Ethics approval

The Animal Care Ethics Committee of the Université Laval approved all *in vivo* experimental protocols (Approval 2021-762). All experiments were performed in accordance with the *Guide to the Care and Use of Experimental Animals* (Canadian Council on Animal Care, 1993, 2nd edition, revised April 2020). Ethics Committee of the Université Laval approved the use of human CSF samples (Approval 2022–2363).

## Availability of data and material

The datasets used and/or analyzed during the current study are available from the corresponding author on reasonable request.

## Author contributions

JPJ, SP and APB designed the study. APB performed all experiments, data analysis and statistical analysis, prepared figures and wrote the manuscript.

JPJ and SP supervised the study and revised the manuscript. All authors agreed to be personally accountable for contributions and ensured that questions related to accuracy and integrity of any part of the work are appropriately investigated, resolved, and documented by literature. All authors read and approved the final manuscript.

## Declaration of competing interest

The authors declare that they have no competing interests. J-P. J. and S. P. are co-inventors of a patent entitled ‘Tdp-43-binding polypeptides useful for the treatment of neurodegenerative diseases (US10202443–B2, WO2016/086320-A1)’.
