# Supplementary material for: Antibody targeting TDP-43 mitigates pathogenic pathways induced by the cerebrospinal fluid of ALS
Source: Neurotherapeutics. 2025 Sep 11;22(6):e00737. doi: 10.1016/j.neurot.2025.e00737 (PMC12664456; doi:10.1016/j.neurot.2025.e00737)
Supplement: Multimedia component 1 [file mmc1.pdf]

Supplemental figures

Supplemental figure 1

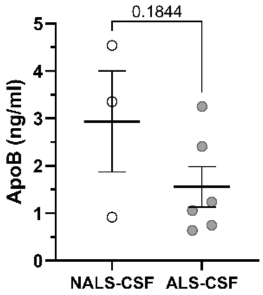

Supplemental fig. 1 ApoB protein concentration in individual sALS (1.557±0.4263; n=6) and control NALS (2.936±1.067; n=3) CSF samples measured by Human ApoB ELISA kit from Invitrogen. Data are shown as mean±SEM

Supplemental figure 2

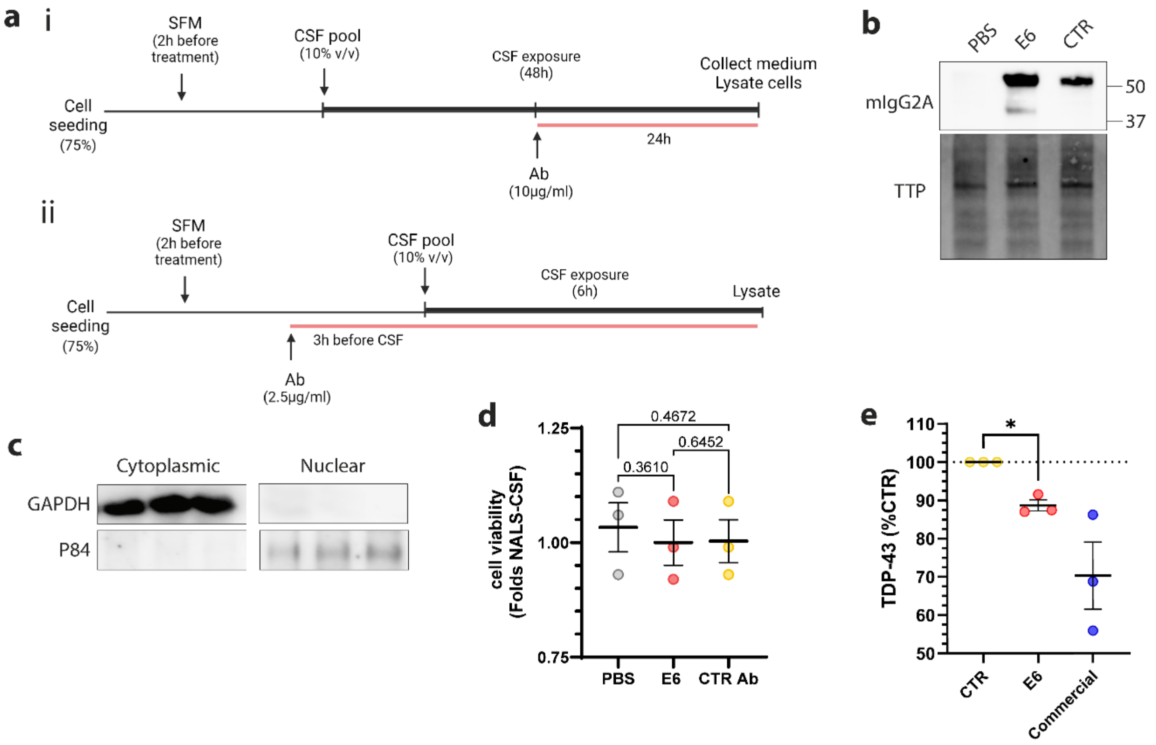

Supplemental fig. 2 (a) Time course of *in vitro* experiments. When cells reached 75% of confluence, medium was changed for serum free medium (SFM) for 2h before CSF or antibody treatments. (i) Time course of treatment for luciferase assay to study the effect of ALS-CSF exposure on NFκB activation in NSC-34-luc-p65 and BV2-luc-p65. Cells were exposed to CSF (10% v/v) for 48h. Additionally, to study the effect of E6 antibody on microglial type of activation, BV2 cells were treated with E6 antibody (10µg/ml), control antibody or PBS. Medium was used for cytokine array (results in Fig. 9d) and lysates were used for blotting (results in Figure 9A-C). Results shown in Figure 9(ii) Time course of treatment for luciferase assay to study the effect of E6 antibody on NFκB activation in BV2-luc-p65 cells (results shown in Figure 8A). Cells were treated with E6 antibody (2.5µg/ml) for 9h and CSF (10% v/v) for 6h. **(b)** Representative blots of lysates from BV2 cells exposed for 24h to E6 antibody (10µg/ml) or equal volume of PBS or control antibody. Membranes were probed with anti-mouse IgG2A HRP-conjugated antibody. Total transferred proteins (TTP) were used as loading reference. **(c)** Representative blots showing cytoplasmic marker GAPDH and nuclear marker P84 in cytoplasmic and nuclear fractions of NSC-34-hTDP-43^{WT} (results shown in Figure 2A). n=3 technical replicates per experiment. **(d)** Cell survival measured by MTS assay of NSC-34-hTDP-43^{WT} cells (n=3 biological replicates) exposed to ALS-CSF for 48h and treated for 24h with E6 antibody (1.000±0.049), control antibody (1.003±0.047) or equal volume of PBS (1.033±0.054). (e) Graph showing TDP-43 levels in ALS-CSF measured by AlphaLISA after overnight incubation followed by immunoprecipitation with E6 antibody (88.38±1.450), control antibody or a commercial antibody directed against TDP-43 (70.31±8.774). Data are

expressed as % of control antibody. Control antibody (CTR Ab): clone 807.33, anti-protein-G of La Crosse virus IgG2A isotype.

Supplemental figure 3

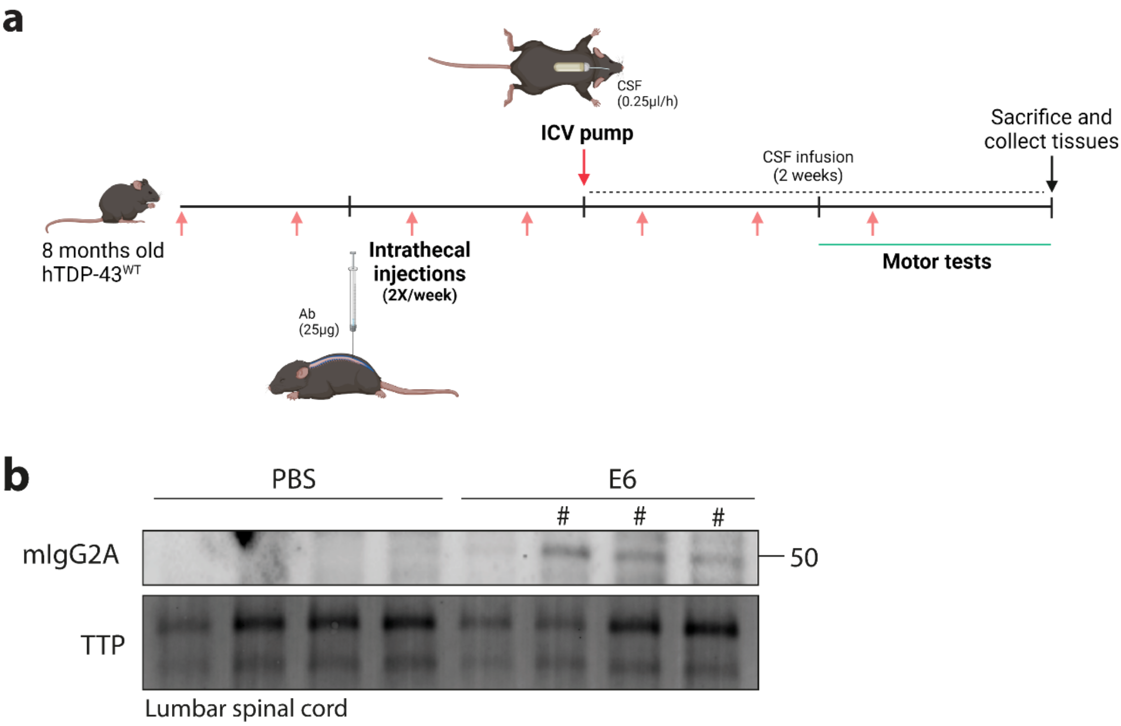

Supplemental fig. 3 Intrathecal administration of E6 antibody. **(a)** Time course of *in vivo* experiments with CSF infusion and IT administration of E6 antibody. ALS-CSF was infused (0.25µl/h) for 2 weeks in 8 months olds hTDP-43^{WT} mice. On week 2, motor performance was tested with gait analysis. Mice were sacrificed 15 days after the pump implantation to collect the tissues. IT administration of the antibody was done by injecting 25µg of E6 antibody or equal volume of PBS in the intrathecal space, twice a week for a total of 7 injections (n=8 mice per condition). Created in BioRender. Poulin-brière, A. (2024) BioRender.com/s97i485 **(b)** Representative Western blot showing E6 antibody heavy chain (50kDa) using an anti-mouse IgG2A on lumbar spinal cord lysates of mice

infused with ALS-CSF and treated i.t. with PBS or E6 antibody (n=4 mice per condition). Total transferred proteins (TTP) were considered as loading reference. (#) highlight the presence of the antibody

Supplemental figure 4

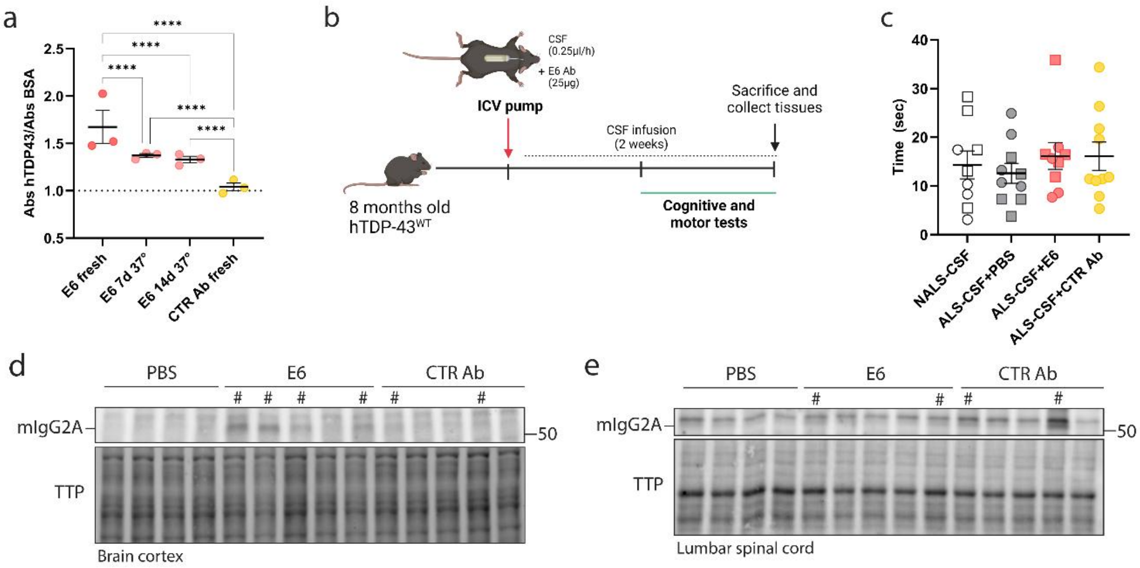

Supplemental fig. 4 Intracerebroventricular administration of E6 antibody. **(a)** Direct ELISA measuring the interaction with recombinant hTDP-43 (0.5µg/ml) of antibodies (E6 antibody or control antibody at 0.4µg/ml) fresh (E6=1.674±0.176, CTR Ab=1.040±0.041) or incubated 7 days (1.372±0.02) and 14 days (1.330±0.034) at 37 ºC . Data are represented as mean± SEM (n=3), and are expressed as ratio of signal (Abs, absorbance) obtained on hTDP43 versus BSA. One-way ANOVA by Tukeys' multiple comparision test ($p<0.0001$). **(b)** Time course of *in vivo* experiments with CSF infusion and ICV administration of E6 antibody. CSF was infused (0.25µl/h) for 2 weeks in 8 months olds hTDP-43^{WT} mice. On week 2, cognitive and motor performances were tested with Novel Object Recognition (NOR), Passive avoidance (PA), grip and gait analysis tests. Mice were sacrificed 15 days

after the pump implantation to collect the tissues. The i.c.v. administration of CSF and antibody was done by putting 25μg of E6 antibody, control antibody or equal volume of PBS in the pump for infusion along with the ALS-CSF (n=10 mice per condition). Created in BioRender. Poulin-brière, A. (2024) BioRender.com/s97i485 **(c)** Total exploration time (seconds) spent exploring both familial and novel objects during day 3 of Novel Object Recognition test for ICV treated mice. Data are represented as mean± SEM. n=10 mice per condition. **(d-e)** Representative western blots showing E6 antibody heavy chain (50kDa) using an anti-mouse IgG2A on **(d)** brain cortex and **(e)** lumbar spinal cord lysates of mice infused with ALS-CSF and 25μg of E6 antibody or control antibody, or equal volume of PBS. n=5 mice per condition. Total transferred proteins (TTP) were considered as loading reference. Control antibody (CTR Ab): clone 807.33, anti-protein G of La Crosse virus IgG2A isotype. (#) highlight the presence of the antibody

Supplemental figure 5

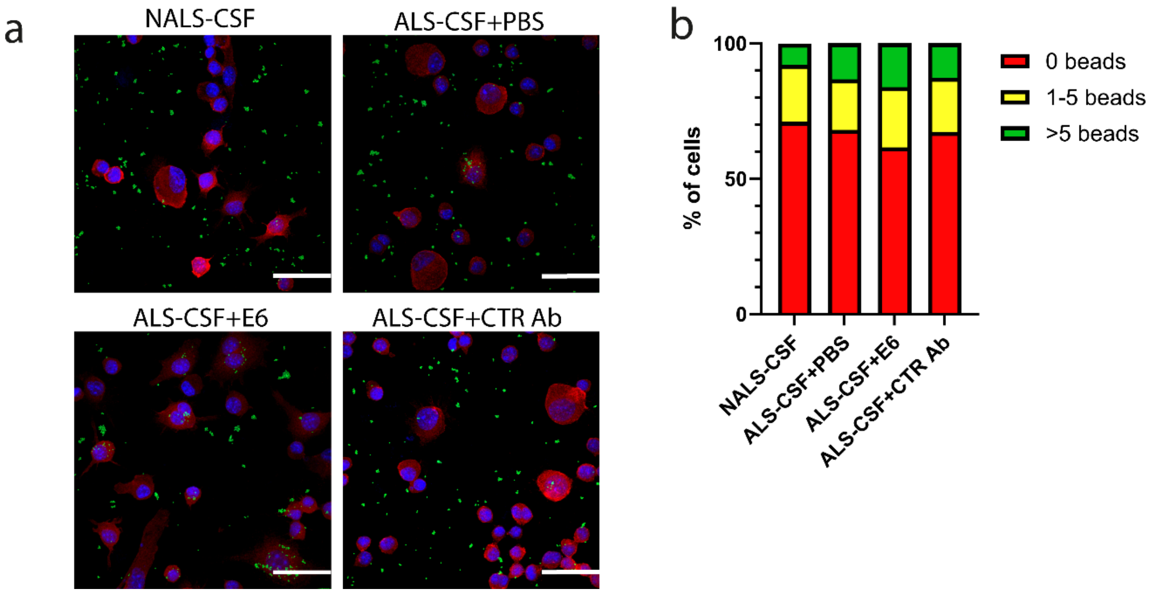

Supplemental fig. 5 (a) Representative images of BV2 cells exposed 48h to CSF (10% v/v) and treated 24h with E6 antibody (10µg/ml), control antibody or PBS, showing phagocytosis ability. Scale bar: 50µm. **(b)** Quantification of the number of beads after phagocytosis assay. At least 500 cells were analysed per condition. Control antibody (CTR Ab): clone 807.33, anti-protein G of La Crosse virus IgG2A isotype

Supplemental methods

Protein extraction methods and blotting assays

Total proteins were obtained from cells using hot SDS 1% as previously described (1). Briefly, samples were homogenized, sonicated, boiled 10min at 98°C and centrifuged at 12000 rpm for 10min. RIPA soluble and insoluble protein fractions were obtained as previously described (2). Nuclear and cytoplasmic proteins were obtained from cells as previously described (3). Briefly, pellet of cells was lysed in RIPA-A (0.3% Triton X-100, 50mM Tris-HCl pH 7.4 and 1mM EDTA and protease inhibitors) and rotated at 4°C 30min. Cytoplasm (supernatant) was obtained after centrifugation at 12000g 4°C for 10min. The nuclei pellet was resuspended in RIPA-B buffer (1% TritonTM X-100, 1% SDS, 50mM Tris- HCl pH 7.4, 500mM NaCl and 1mM EDTA) containing protease inhibitors cocktail (Roche). Nuclear fraction (supernatant) was obtained after centrifugation at 12000g 4°C for 10min.

Brain cortices or lumbar spinal cords from mice were processed to obtain total, soluble and insoluble fractions as follows. Tissues were homogenized in cold NP-40 lysis buffer (20-mM Tris-HCl pH 7.4, 150-mM NaCl, 10% glycerol, 1% NP-40, 5-mM EDTA, 1-mM DTT and protease inhibitors cocktail). Lysate of each sample was divided in two tubes. One tube was supplemented with SDS (1,5%) and urea (3M) and considered as the total protein fraction. The second tube was used for soluble and insoluble fractionation. Lysates were rotated for 30min at 4°C and then centrifuged for 20min at 15800g at 4°C. Supernatants were used as soluble fraction. Pellet was washed once with NP-40 lysis buffer and sonicated in 6-M urea and 3% SDS. NP-40 buffer supplemented with protease and phosphatase inhibitor cocktail. SDS-Urea lysates were collected as insoluble fraction.

Proteins were quantified using spectrophotometer (Epoch 2, Biotek). Twenty micrograms of proteins were loaded and resolved in 10% stain-free polyacrylamide SDS-PAGE gel (Bio-Rad) and transferred on PVDF membranes (Immobilon-P, Millipore) after stain-free gel activation by 1min UV exposure. Western blot was performed by blocking in 5% BSA (BioBasic Canada) prepared in TBS-tween 1% (VWR Lifescience) (TBS-T). Proteins of interest were detected by incubating the membrane overnight in blocking buffer with primary antibodies (Table 2). Finally, membranes were incubated with respective fluorophore-tagged or HRP-tagged secondary antibodies (Table 2) in 2,5% BSA blocking buffer or TBS-T. Chemiluminescence was revealed by electrogenerated chemiluminescence (ECL) reagent (Thermo Fisher Scientific) and a ChemiDoc MP Imaging System (Bio-Rad). Immunoreactivity was quantified by Image Lab software (Bio-Rad) and normalized on Total Transferred Proteins (TTP) acquisition of the stain-free signal.

ELISA

The ability of E6 antibody to recognize TDP-43 after incubation at 37ºC for 7 or 14 days was measured using direct ELISA kit (Preprotech). 0.05µg of recombinant human TDP-43 (rhTDP-43) (R&D sytems), or BSA (New England BioLabs) as a control, were loaded onto ELISA plate and incubated overnight at room temperature. The following day, the wells were washed 4 times with washing buffer and incubated for 2 hours with 0.4µg/ml monoclonal antibodies (MédiMabs, Abnova) diluted in PBS. The wells were then incubated for 2 hours with anti-mouse-HRP-conjugated antibody (1: 10 000) (Table 2). Chemiluminescence was read at 450nm using an EnSpire 2300 Multilabel Reader (PerkinElmer).

Phagocytosis assay

Phagocytic capacity of microglial cells was examined as described previously (4). Briefly, BV2 cells were exposed to ALS-CSF and E6 or control antibody as described above. Cells were then exposed to the beads (aqueous green, fluorescent latex beads of 1 μm diameter. Sigma, #L1030) at a concentration of 1:100 in DMEM-F12 (beads 0.01% v/v, 0.05% FBS v/v). After 1 h of incubation, cells were fixed with PFA 4% and immunostaining for Iba-1 and DAPI was performed. Pictures were acquired at a high magnification of 60X. For each condition, at least 20 pictures and at least 500 cells were analysed. We counted the number of cells containing 0 beads, between 1 and 5 beads, and more than 5 beads.

MTS assay

MTS assay was performed under cell culture conditions and treatment paradigm used for luciferase assay and performed as per the manufacturer's instructions (Promega, Madison). Briefly, after NSC-34-hTDP-43^{WT} exposure to CSF and treatment, cell culture medium was transferred to a 96w/p in duplicates and MTS reagent was added to a ratio of 2:10 and incubated for 1h at 37ºC. The absorbance was determined at 490 nm using an EnSpire 2300 Multilabel reader (Perkin Elmer, Waltham, MA, USA).

References

1. Pozzi S, Thammisetty SS, Julien JP. Chronic Administration of Pimozide Fails to Attenuate Motor and Pathological Deficits in Two Mouse Models of Amyotrophic Lateral Sclerosis. Neurotherapeutics. 2018;15(3):715-27.
2. Iguchi Y, Eid L, Parent M, Soucy G, Bareil C, Riku Y, et al. Exosome secretion is a key pathway for clearance of pathological TDP-43. Brain. 2016;139(12):3187-201.
3. Lauranzano E, Pozzi S, Pasetto L, Stucchi R, Massignan T, Paolella K, et al. Peptidylprolyl isomerase A governs TARDBP function and assembly in heterogeneous nuclear ribonucleoprotein complexes. Brain. 2015;138(Pt 4):974-91.
4. Barreto-Núñez R, Béland LC, Boutej H, Picher-Martel V, Dupré N, Barbeito L, et al. Chronically activated microglia in ALS gradually lose their immune functions and develop unconventional proteome. Glia. 2024;72(7):1319-39.
